# Family correlates of physical activity and parent-rated motor competence in preschool children from a socioecological perspective: a cross-sectional analysis of 10,967 children in China

**DOI:** 10.3389/fpsyg.2026.1898292

**Published:** 2026-10-21

**Authors:** Yanfeng Zhang, Xiang Pan, Yibo Gao, Mingzhe Li, Lupei Jiang, Deqiang Zhao, Jiaxin Chen, Chunmiao Wang, Xueli Zhao, Haixia Hu

**Affiliations:** 1 China Institute of Sport Science, Beijing, China; 2 Graduate School of Health and Sports Science, Juntendo University, Inzai, Chiba, Japan; 3 Department of Psychology, Qingdao University, Qingdao, China; 4 Weifang Aiyoudong Children and Youth Sports Health Research Institute, Weifang, China

**Keywords:** China, developmental psychology, family environment, motor competence, parental support, physical activity, preschool children, social cognitive theory

## Abstract

Family correlates of preschool children’s physical activity (PA) and motor competence are seldom compared within a single adjusted model. We designed and conducted a national household survey across all 31 province-level divisions of China in 2020 using three-stage probability-proportional-to-size sampling and included 10,967 children aged 3–6 years. Caregivers reported parental support, parenting orientation, exercise, screen time, co-activity, parental obesity, home sport equipment, neighborhood environment, and child temperament, together with daily PA and parent-rated motor competence. Parental support combined attitudes and enacted support in a six-item scale (Cronbach’s alpha = 0.76). Survey-weighted regressions with county sampling units nested in province strata estimated semistandardized associations. A cross-sectional decomposition used the percentile bootstrap resampling of sampling units; multiple imputation and inverse-probability weighting were used to assess missing-data sensitivity. Parental support was associated with parent-rated motor competence (0.32 points per standard deviation [SD], 95% confidence interval [CI] 0.26–0.38). With two items overlapping co-activity removed, this estimate was 0.22 (0.17–0.28), and contrasts with sociability and equipment no longer excluded zero. Child sociability, home sport equipment, and caregiver co-activity were associated with PA (8.3, 8.0, and 5.9 min/day per SD). In the decomposition, 40 percent of the support–PA association was statistically accounted for by home equipment, co-activity, and maternal exercise; the majority of the support–competence association was not accounted for by these behaviors. Parental obesity was not associated with either outcome. These results describe adjusted associations conditional on the measures and covariates used and identify family and child characteristics for further study with independently assessed outcomes.

## 1 Introduction

Physical activity (PA) and motor competence in early childhood are foundational for later health, movement skills, and the formation of activity habits (World Health Organization, 2019; Carson et al., 2017). The majority of preschoolers fall well short of activity guidelines across diverse national settings (Abdeta et al., 2025; Mwase-Vuma et al., 2025), so the family-level correlates of young children’s activity and competence are a practical priority for both surveillance and intervention design. At this age, the home is the primary waking context, and caregivers structure the majority of the opportunities for movement and for emerging motor skills.

Three parental domains recur in the preschool literature. Parental support for activity, expressed through encouragement, role-modeling intention, and the valuation of activity, represents a cognitive and motivational antecedent of caregiver behavior (Beets et al., 2010). Parental exercise behavior is a behavioral expression that children can observe and may imitate, consistent with social cognitive theory and with documented parent–child activity concordance and role modeling (Bandura, 1986; Davison et al., 2003; Webber and Loescher, 2013; Yao and Rhodes, 2015). Parental weight status has been proposed as a transmission channel through shared genes and lifestyles and has informed interventions that target parental body mass as a proxy for child risk (Whitaker et al., 1997).

Two gaps limit the usefulness of this evidence. First, the three domains are rarely examined together, and the wider family system, including parenting orientation, parental sedentary behavior, direct co-activity, the home material environment, the neighborhood, and child temperament, is seldom modeled alongside the domains (Dowda et al., 2011; Patnode et al., 2010; Sleddens et al., 2012); when correlates are studied one at a time, it is impossible to know which has an adjusted association, or whether apparently important factors are confounded by one another (Sallis et al., 2000; Yao and Rhodes, 2015). Second, PA and motor competence are usually treated as interchangeable child outcomes, yet they may have different correlates: activity volume reflects daily opportunity and enabling resources, whereas motor competence is a more slowly accruing developmental attribute with its own family and environmental determinants (Barnett et al., 2016; Iivonen and Sääkslahti, 2014; Robinson et al., 2015; Stodden et al., 2008). Whether the same parental factors are associated with both outcomes and how those associations relate to measured parental behaviors has not been characterized in a large non-Western sample.

Contemporary Chinese preschoolers grow up amid the legacies of the one-child policy, a cultural emphasis on academic preparation alongside holistic development, and rapid urbanization that has reduced outdoor access (Cameron et al., 2013). The family environment has been linked to children’s fitness and activity outcomes in Chinese national samples (Pan et al., 2022, 2023), yet locally validated instruments for caregiver support directed at child activity have only recently become available, and nationally representative evidence comparing adjusted associations of parental factors in young Chinese children remains scarce (Pan et al., 2025c).

In a nationally representative sample of 10,967 Chinese 3- to 6-year-olds, we pursued three objectives. The first was to identify, when an enriched set of parental, family-environment, and child factors are modeled together, how these factors are associated with children’s PA and with their parent-rated motor competence. The second was to determine whether the patterns of association differ between the two outcomes. The third was to quantify the extent to which the association between parental support and child outcomes can be statistically decomposed through observable parental behaviors. These objectives concern cross-sectional associations among caregiver-reported measures.

## 2 Methods

### 2.1 Study design and participants

We designed and conducted the 2020 round of the China National Fitness Activity Status Survey, and this study analyses the sample of children aged 3–6 years. The survey is the national fitness surveillance program of mainland China; our group wrote the protocol and the child questionnaire, trained the provincial field teams, and ran fieldwork across all 31 provincial-level divisions between September and November 2020. We used a three-stage stratified probability-proportional-to-size design. In the first stage, we built a county-level sampling frame from the sixth national population census and drew 10–20 county-level units within each province with probability proportional to population size, stratifying by the level of socioeconomic development and taking geographic location within the province into account; 486 county-level units were selected nationally. In the second stage, we built a frame of all residential committees and village committees in each selected county, fixed the number of urban and rural committees from that county’s urban–rural population ratio, and drew 13 committees; the urban–rural classification carried in the data file accordingly describes the sampled committee and not the county. In the third stage, we drew from each committee a fixed quota of respondents, including two children aged 3–6 years, with a parent or primary caregiver serving as the proxy respondent for these children. Our trained surveyors administered the questionnaire through on-site guided completion during home visits, and we carried out electronic upload, double data entry, and telephone verification under the multi-level quality-control procedure. The 3- to 6-year-old sample comprises 10,967 children from 470 of these county-level units; the remaining units included no children of this age in the file. Each record includes the analytic weight we constructed for the survey, which is constant within province-by-urban–rural residence by child sex cells and is normalized to a mean of 1; this weight was applied in all analyses. Daily PA could not be scored for 1,302 children whose caregiver answered “cannot say” for at least one weekday segment, and after listwise deletion on the 19 predictors the analytic samples comprised 8,059 children for PA and 8,824 for motor competence (participant flow in Supplementary Figure S1; variable-specific missingness and a comparison of included and excluded children in Supplementary Tables S5, S6).

### 2.2 Measures

Daily average PA (minutes per day) was derived from six caregiver-reported duration items. For weekdays, caregivers reported the child’s total physical activity time, inside and outside kindergarten, in three segments of the day (waking to noon, noon to 6 p.m., and 6 p.m. to bedtime), each on a six-category scale (no activity; 1–15; 16–30; 31–60; more than 60 min; cannot say). For weekend days, caregivers reported out-of-home physical activity time, including movement-based classes, in the same three segments on a five-category scale (1–15, 16–30, 31–60; more than 60 min; no out-of-home activity). Categories were converted to interval midpoints (8, 23, and 45 min), the two zero categories to 0, and the open-ended category to 75 min; “cannot say” was treated as missing. Segment values were summed within day type and combined as (weekday sum × 5 + weekend sum × 2)/7, giving a possible range of 0–225 min per day. The full item wording, the weighted distribution of every response category, and sensitivity analyses that assign 90 or 120 min to the open-ended category are in Supplementary Tables S1, S7. Parent-rated motor competence was the sum of three items comparing the child with same-age peers on overall athletic ability, speed of learning new movements, and a strength-endurance-agility composite (Cronbach’s alpha = 0.81; range 3–15); it is a caregiver rating, not an observed motor test. For the secondary adiposity analysis, height and weight reported by the caregiver were converted to BMI and expressed as an internally standardized index (the residual from a regression of BMI on age in months and sex, standardized to mean 0 and standard deviation [SD] 1 within the sample); this index is not a growth-reference *z*-score and is analyzed only in the Supplement.

Parental support for the child’s activity was measured with a six-item scale we developed for this surveillance program through a three-round Delphi process with 83 senior experts from provincial sport authorities (item content in Supplementary Table S4). Two items describe beliefs (parents should set an example; activity is important and should be sustained over the long term); two describe encouragement and therefore refer to what caregivers do as well as to what they endorse (encouraging outdoor play in good weather; encouraging enrollment in organized sport); and two describe enacted support (taking the child to cultural and sport activities; exercising or playing actively with the child at home). The scale is therefore a measure of parental support for the child’s activity and is not interpreted as an attitude measure. Items were rated on five-point agreement scales and averaged, scored so that higher values indicate more support (Cronbach’s alpha = 0.76); a scale score required all six items. Because the two enacted-support items overlap conceptually with caregiver co-activity, all analyses were repeated with a four-item reduced-overlap score (Cronbach’s alpha = 0.74); items 1 and 4 of that reduced score describe encouragement, which refers to what caregivers do as well as to what they endorse, so the construct encompasses both endorsement and supportive practices. A detailed psychometric evaluation of the instrument, including factor structure and measurement invariance, is reported separately.^1^ Additional parental and family measures were parenting orientation (a nominal item with three options: preference-led, weakness-targeted, or knowledge-based selection of activities; entered as two indicator variables with preference-led as the reference), persistence when a child resists (three ordered options from letting the child stop to insisting on continued participation; entered as a linear term), perceived child weight (five options collapsed to heavier than right, about right, or thinner than right; entered as two indicators with about right as the reference), father and mother exercise frequency (five ordered categories from more than three times a week to never; “unclear or not applicable” treated as missing), father and mother weekend screen time (five ordered categories from under 1 to 4 h or more per day; “cannot say” treated as missing), caregiver weekend co-activity time (seven options from no time to 12 h or more, coded 0–6), parental obesity (a yes/no item answered separately for the father and the mother), a count of sport-related items among the play equipment provided at home (0–10), and perceived neighborhood sport environment (five items, averaged). Child temperament was indexed by a caregiver-reported sociability composite (three items on quietness, disliking company, and preferring to be alone, reverse-scored, and averaged). Covariates were child age in months, sex, urban–rural residence, household income (ten ordered bands), and the higher of paternal and maternal education (five ordered levels). A variable dictionary with the source item, coding, and range of every variable is provided as Supplementary Table S8.

### 2.3 Statistical analysis

All analyses were design-based. Variance estimation used Taylor linearization with the county-level unit as the primary sampling unit nested within province strata (the ultimate-cluster approximation to the three-stage design), the survey weight described above, and t reference distributions with design degrees of freedom (number of sampling units minus number of strata, 438 for PA and 439 for motor competence). The first-stage strata, level of socioeconomic development, and geographic location within the province, are not carried in the analysis file, so the province is used as the stratum; ignoring finer stratification is the conservative choice. The urban–rural classification is applied by the design at the second stage to fix the number of urban and rural committees within a selected county and therefore describes a committee-level attribute: it varies within 405 of the 470 county-level units, does not partition the primary sampling units, and is included in the models as a respondent-level covariate. It is not used to define strata. A sensitivity analysis that assigns each county its majority classification and stratifies by province and urban–rural residence is reported in Supplementary Table S14. Main models were survey-weighted linear regressions for PA and for motor competence with all 19 predictors entered simultaneously. To place predictors on a comparable footing, continuous and ordinal predictors were centered and scaled to the analytic-sample SD, whereas binary and nominal predictors were entered as indicators; the resulting coefficients are semistandardized (outcome in its natural units, continuous predictors per SD, indicators per category contrast) and are reported with exact 95% confidence intervals (CI). Coefficients per natural unit and the predictor SDs are given alongside so that either scale can be read. Selected differences between semistandardized coefficients were tested directly as linear contrasts. The share of model R-squared assigned to each predictor was estimated by the Lindeman–Merenda–Gold (LMG) averaging-over-orderings method with the five covariates always included. These comparisons describe the fitted associations and depend on the included variables and their measurement. Multicollinearity was assessed by variance inflation factors. Because inference at the province level relies on 31 clusters, the main models were re-estimated with province-clustered standard errors using the CR2 small-sample adjustment and Satterthwaite degrees of freedom. Missing data were handled by listwise deletion in the main models and by two sensitivity approaches: multiple imputation by chained equations (20 imputed data sets, predictive mean matching, estimates pooled by Rubin’s rules with the design applied to each imputed data set) and inverse-probability weighting. The imputation model contained both outcomes, all 19 predictors, the survey weight, indicators for the 31 provincial strata, and the county-level means of the analysis variables, so that the stratification and the between-cluster variation of the design were represented in the imputation as well as in the analysis. For inverse-probability weighting, the survey weight was multiplied by the inverse of the predicted probability of being a complete case from a logistic model containing child age, sex, urban–rural residence, household income, parental education, province indicators, and every remaining predictor that was at least 99 percent complete, including the three variables on which included and excluded children differ most (parental support, home sport equipment, and co-activity); the model is reported in Supplementary Table S15, and the five-covariate model used previously is retained alongside it for comparison. Because the variables that drive exclusion are themselves the missing ones, both sensitivity analyses assume missingness at random conditional on the modeled variables; selection on unmeasured factors remains unaddressed.

Curvature in the two associations of interest was examined by adding a quadratic term and confirmed with a natural cubic spline (3 degrees of freedom); adjusted predictions with CIs at the 10th, 50th, and 90th percentiles of the predictor are reported. The association of support with each outcome was decomposed through four measured behavioral variables (home sport equipment, co-activity, maternal exercise, and paternal exercise) using the product-of-coefficients approach in the same analytic sample as the main model, adjusting for the five covariates; the total association, the direct association, and all four indirect components are reported per one scale point of support, with 95% percentile bootstrap confidence intervals from 1,000 bootstrap resamples in which county-level units were resampled with replacement within province strata and the survey weight was retained. Six interactions of support with child sociability, sex, urban–rural residence, income, parental education, and age were tested for each outcome (12 tests) with Benjamini–Hochberg correction; none were pre-registered, and all are reported as exploratory. Analyses were conducted in R 4.4.2 (packages survey, mice, mitools, clubSandwich, and relaimpo) by the analyzing author; point estimates of the main models were cross-checked against a Python implementation (statsmodels). The analysis code is provided in Supplementary Data S1. Because the design is cross-sectional and exposures and outcomes were reported by the same caregiver, all estimates are interpreted as associations.

## 3 Results

The weighted sample (*N* = 10,967) was 54.2 percent boys and 61.4 percent urban, with a mean age of 65.8 months (SD 14.0); 3,925 children were younger than 60 months, 6,876 were 60 months or older, and 166 had no usable birth date. Weighted daily PA averaged 114.5 min (SD 56.0) among the 9,665 children for whom it could be scored, and mean parent-rated motor competence was 11.4 (SD 2.1) on the 3-to-15 scale. Caregivers reported 23.7 percent of fathers and 14.0 percent of mothers as obese. Parental support averaged 4.2 on the 1-to-5 scale, and parental exercise frequency was moderate for both parents. Full characteristics by sex, residence, and age group are presented in Table 1.

**Table 1:** Weighted participant characteristics (*N* = 10,967).

Variable	Overall	Boys	Girls	Urban	Rural	3–4 year	5–6 year
*N* (unweighted)	10,967	5,832	5,135	6,993	3,972	3,925	6,876
Child age, months	65.8 (14.0)	65.9 (14.1)	65.6 (13.9)	65.5 (14.0)	66.3 (13.9)	50.5 (5.6)	74.4 (9.0)
Boys, %	54.2	100.0	0.0	54.6	53.7	54.3	54.4
Urban, %	61.4	61.8	60.9	100.0	0.0	63.0	60.6
Daily PA, min/day	114.5 (56.0)	114.7 (56.0)	114.2 (55.9)	116.1 (55.3)	111.9 (57.0)	118.1 (57.6)	112.6 (54.9)
Parent-rated motor competence (3–15)	11.4 (2.1)	11.5 (2.1)	11.4 (2.1)	11.5 (2.1)	11.3 (2.2)	11.5 (2.1)	11.4 (2.1)
Parental support for child activity (1–5)	4.2 (0.6)	4.2 (0.6)	4.2 (0.6)	4.3 (0.6)	4.1 (0.6)	4.2 (0.6)	4.2 (0.6)
Father’s exercise frequency (1–5)	3.4 (1.3)	3.5 (1.3)	3.4 (1.3)	3.5 (1.3)	3.3 (1.4)	3.5 (1.3)	3.4 (1.3)
Mother’s exercise frequency (1–5)	3.5 (1.3)	3.5 (1.3)	3.5 (1.3)	3.5 (1.3)	3.4 (1.4)	3.5 (1.3)	3.4 (1.3)
Father obese, %	23.7	23.3	24.2	25.0	21.5	24.2	23.5
Mother obese, %	14.0	13.6	14.6	14.2	13.8	15.2	13.5
Father screen time (1–5)	2.4 (1.3)	2.4 (1.3)	2.4 (1.3)	2.5 (1.3)	2.3 (1.2)	2.4 (1.3)	2.4 (1.3)
Mother screen time (1–5)	2.3 (1.2)	2.3 (1.2)	2.3 (1.2)	2.3 (1.2)	2.2 (1.1)	2.3 (1.2)	2.2 (1.2)
Caregiver co-activity (0–6)	2.1 (1.3)	2.1 (1.3)	2.1 (1.3)	2.2 (1.3)	1.9 (1.3)	2.2 (1.3)	2.1 (1.3)
Home sports equipment (0–10)	1.8 (1.6)	1.9 (1.6)	1.7 (1.6)	1.9 (1.6)	1.6 (1.6)	1.6 (1.5)	1.9 (1.6)
Neighborhood sports environment (1–5)	3.9 (0.8)	3.9 (0.8)	3.9 (0.8)	3.9 (0.8)	3.8 (0.8)	3.9 (0.8)	3.9 (0.8)
Child sociability (1–5)	3.5 (0.8)	3.5 (0.8)	3.4 (0.8)	3.5 (0.8)	3.4 (0.8)	3.5 (0.8)	3.5 (0.8)
Persistence when child resists (1–3)	2.0 (0.6)	2.0 (0.6)	2.0 (0.6)	2.0 (0.6)	2.0 (0.7)	2.0 (0.6)	2.0 (0.6)
Household income (1–10)	3.7 (2.0)	3.7 (2.0)	3.6 (2.0)	4.1 (2.0)	3.0 (1.7)	3.8 (2.0)	3.6 (2.0)
Highest parental education (1–5)	3.0 (0.9)	3.0 (0.9)	3.0 (0.9)	3.3 (0.9)	2.6 (0.9)	3.1 (0.9)	2.9 (0.9)
Parenting orientation: Preference-led, %	63.8	63.0	64.7	63.7	64.0	65.7	62.8
Parenting orientation: Weakness-targeted, %	21.4	21.6	21.2	22.0	20.5	20.0	22.2
Parenting orientation: Knowledge-based, %	14.8	15.4	14.1	14.3	15.5	14.4	15.0
Perceived child weight: About right, %	73.6	73.1	74.0	73.0	74.4	74.9	72.9
Perceived child weight: Heavier, %	7.4	7.5	7.3	7.3	7.6	7.3	7.3
Perceived child weight: Thinner, %	19.0	19.3	18.7	19.7	18.0	17.7	19.8

Values are weighted means (SD) for continuous variables and weighted percentages for binary and categorical variables; *N* is the unweighted count of children in each column. Age subgroups are split at 60 months; 166 children with missing age are included in the other columns. The survey weight is constant within province-by-urban–rural-by-sex cells.

### 3.1 Adjusted associations by outcome

In the fully adjusted models (Table 2; Figure 1), the pattern of adjusted associations differed by outcome. All values below are semistandardized coefficients: the change in the outcome per one SD of a continuous predictor, or the contrast for an indicator, on each outcome’s own scale. For parent-rated motor competence (*R*^2^ = 0.104; scale 3–15 points), the parental support coefficient was 0.32 points per SD (95% CI 0.26–0.38). Direct contrasts indicated a larger coefficient for support than for child sociability (0.19, 95% CI 0.13–0.25; difference 0.13, 95% CI 0.04–0.21, *p* = 0.005) and home sports equipment (0.18, 95% CI 0.13–0.23; difference 0.14, 95% CI 0.06–0.22, *p* = 0.001); the neighborhood sport environment coefficient was 0.16 (95% CI 0.11–0.22). With the four-item reduced-overlap support score, which omits the two enacted-support items, the support coefficient fell by approximately 30 percent to 0.22 points per SD (95% CI 0.17–0.28). The corresponding coefficients were similar for neighborhood sport environment (0.20, 95% CI 0.15–0.26), home sports equipment (0.19, 0.14–0.24), and child sociability (0.19, 0.13–0.25), with contrasts with sociability of 0.04 (95% CI −0.05 to 0.12, *p* = 0.43) and with home equipment of 0.04 (−0.05 to 0.12, *p* = 0.40; Supplementary Table S12). The contrasts involving support therefore depended on the support measure; neither contrast with the reduced-overlap score excluded zero. Among the indicator variables, a weakness-targeted parenting orientation (−0.26 points versus preference-led, 95% CI −0.39 to −0.14) and a child perceived as thinner than right (−0.36 versus about right, 95% CI −0.50 to −0.23) were associated with lower scores. For physical activity (*R*^2^ = 0.115; minutes per day), child sociability (8.3 min/day per SD, 95% CI 6.7–9.9) and home sports equipment (8.0, 95% CI 6.5–9.5) had positive coefficients, and their contrast did not exclude zero (difference 0.31, 95% CI −1.98 to 2.59, *p* = 0.79); the coefficients for caregiver co-activity and parental support were 5.9 (95% CI 4.4–7.4) and 4.5 (95% CI 2.8–6.1), respectively, with no significant difference between them (*p* = 0.22). Father and mother screen time were positively associated with PA (3.8 and 2.8 min/day per SD; *p* < 0.001 and *p* = 0.003). In the relative-importance analysis, sociability, equipment, and co-activity accounted for 27, 27, and 17 percent of the *R*^2^ assigned to the focal predictors for PA, and parental support accounted for 34 percent for motor competence (Supplementary Table S11). Coefficients are comparable within an outcome but not across outcomes. Variance inflation factors did not exceed 1.8. Parental obesity was not associated with either outcome (PA: father −0.2 min/day, 95% CI −3.3 to 2.9; mother 3.1, 95% CI −1.1 to 7.2; motor competence: father 0.00, 95% CI −0.11 to 0.12; mother 0.02, 95% CI −0.13 to 0.17). Province-clustered inference with the CR2 small-sample correction agreed with the design-based inference for every coefficient above; the only coefficient whose inference depended on the clustering choice was maternal exercise frequency for PA (2.1 min/day per SD; design-based *p* = 0.014, province-clustered *p* = 0.11; Supplementary Table S9).

**Table 2:** Fully adjusted semistandardized associations of parental, family-environment, and child factors with physical activity and parent-rated motor competence.

Predictor (unit of change)	PA, min/day: *b* (95% CI)	*p*	Parent-rated motor competence, points: *b* (95% CI)	*p*	Predictor SD (PA/motor sample)
Perceived child weight: thinner than right (vs. about right)	−2.61 (−5.95, +0.73)	0.125	−0.363 (−0.497, −0.230)	<0.001	–
Parental support (per SD)	+4.45 (+2.80, +6.10)	<0.001	+0.318 (+0.258, +0.378)	<0.001	0.56/0.56
Parenting orientation: weakness-targeted (vs. preference-led)	+2.28 (−1.61, +6.16)	0.250	−0.265 (−0.387, −0.142)	<0.001	–
Child sociability (per SD)	+8.31 (+6.70, +9.92)	<0.001	+0.191 (+0.133, +0.249)	<0.001	0.79/0.79
Home sports equipment (per SD)	+8.01 (+6.47, +9.55)	<0.001	+0.177 (+0.126, +0.228)	<0.001	1.61/1.61
Neighborhood sport environment (per SD)	+0.41 (−1.25, +2.08)	0.627	+0.163 (+0.109, +0.216)	<0.001	0.76/0.77
Perceived child weight: heavier than right (vs. about right)	−4.55 (−9.62, +0.53)	0.079	−0.136 (−0.395, +0.123)	0.303	–
Urban (vs. rural)	−0.57 (−3.77, +2.64)	0.728	−0.118 (−0.235, −0.002)	0.047	–
Father screen time (per SD)	+3.81 (+1.87, +5.75)	<0.001	−0.092 (−0.165, −0.018)	0.014	1.25/1.26
Father exercise frequency (per SD)	+0.45 (−1.09, +1.98)	0.569	+0.077 (+0.013, +0.140)	0.018	1.24/1.26
Child age (per SD)	−2.73 (−4.16, −1.30)	<0.001	−0.066 (−0.116, −0.016)	0.010	14.00/14.04
Household income (per SD)	+1.01 (−0.61, +2.63)	0.222	+0.062 (−0.001, +0.126)	0.054	2.05/2.06
Mother exercise frequency (per SD)	+2.08 (+0.43, +3.74)	0.014	+0.053 (−0.010, +0.117)	0.098	1.24/1.27
Mother screen time (per SD)	+2.84 (+0.99, +4.70)	0.003	−0.046 (−0.112, +0.021)	0.177	1.17/1.18
Boy (vs. girl)	−0.95 (−3.47, +1.56)	0.458	+0.041 (−0.049, +0.130)	0.370	–
Caregiver co-activity (per SD)	+5.90 (+4.40, +7.41)	<0.001	+0.033 (−0.024, +0.091)	0.258	1.25/1.26
Parenting orientation: knowledge-based (vs. preference-led)	+2.17 (−2.20, +6.54)	0.330	+0.032 (−0.102, +0.166)	0.640	–
Highest parental education (per SD)	−1.39 (−3.25, +0.46)	0.140	+0.030 (−0.036, +0.096)	0.366	0.95/0.95
Mother obese (vs. not)	+3.08 (−1.06, +7.22)	0.145	+0.021 (−0.129, +0.170)	0.786	–
Persistence when child resists (per SD)	−0.32 (−1.62, +0.97)	0.624	−0.012 (−0.062, +0.038)	0.642	0.60/0.60
Father obese (vs. not)	−0.18 (−3.28, +2.93)	0.911	+0.002 (−0.113, +0.117)	0.970	–

Survey-weighted linear regression with all predictors entered simultaneously; design-based standard errors (county sampling units within province strata; design df = 438 for PA, 439 for motor competence). Continuous and ordinal predictors are scaled to the analytic-sample SD (coefficient = change in outcome per 1 SD of the predictor); binary and nominal predictors are entered as indicators (coefficient = difference from the reference category). Coefficients are comparable within an outcome but not across outcomes. PA: *N* = 8,059, *R*-squared = 0.115; motor competence: *N* = 8,824, *R*-squared = 0.104. Rows are displayed in absolute-coefficient order for parent-rated motor competence; this display order does not establish differences between coefficients. Coefficients per natural unit, VIFs, province-clustered, and missing-data sensitivity estimates are in Supplementary Tables S9, S10.

**Figure 1:**
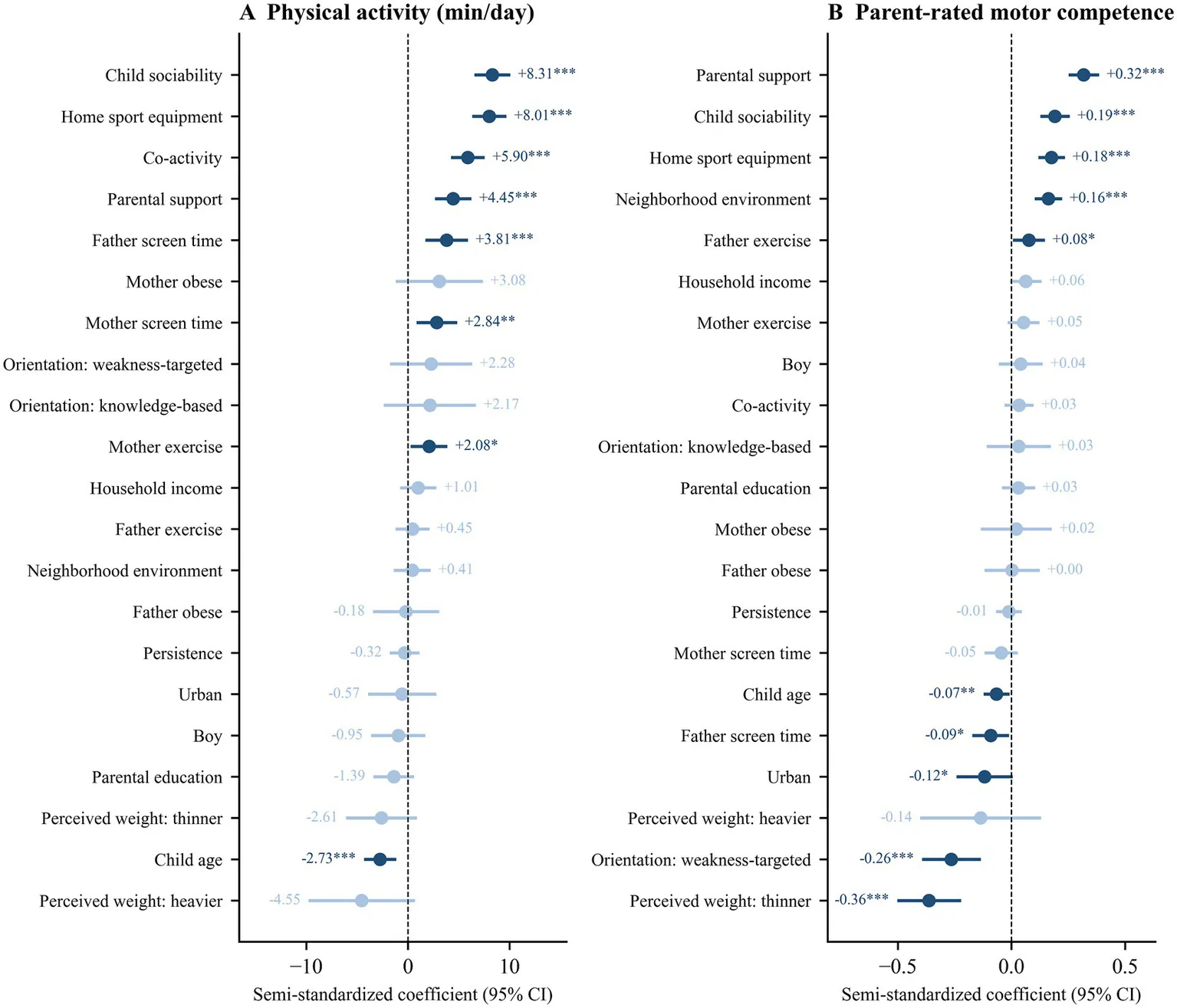
Fully adjusted semistandardized associations of family and child factors with preschoolers’ physical activity and parent-rated motor competence. Forest plots of coefficients from survey-weighted linear regression with design-based 95% confidence intervals (county sampling units nested in province strata). Continuous predictors are per SD; indicator variables are contrasts against the reference category named in Table 2. **(A)** Physical activity, in minutes per day (*N* = 8,059); **(B)** parent-rated motor competence, on the 3–15 scale (*N* = 8,824). Predictors are displayed in coefficient order, with estimates printed beside each marker; this display order does not establish differences between coefficients. Filled markers indicate *p* < 0.05, and grey markers indicate *p* ≥ 0.05. Coefficients are comparable within a panel but not across panels.

### 3.2 Curvature

Both associations examined for curvature departed modestly from linearity without reversing direction within the observed range, and a natural-spline fit confirmed monotonicity in both cases (Figure 2; Supplementary Table S2). The association of parental support with motor competence was slightly convex (quadratic term 0.20, *p* = 0.001): adjusted motor competence was 10.80 points (95% CI 10.69–10.90) at the 10th percentile of support (3.50 on the 1–5 scale), 11.48 (11.41–11.56) at the median (4.33), and 12.24 (12.12–12.36) at the 90th percentile (5.00). The association of co-activity with PA was concave (quadratic term −1.02, *p* = 0.004): adjusted PA was 105.2 min/day (95% CI 102.4–108.0) at the 10th percentile of co-activity (1 on the 0-to-6 scale), 115.7 (113.5–117.9) at the median (2), and 130.5 (127.5–133.6) at the 90th percentile (4).

**Figure 2:**
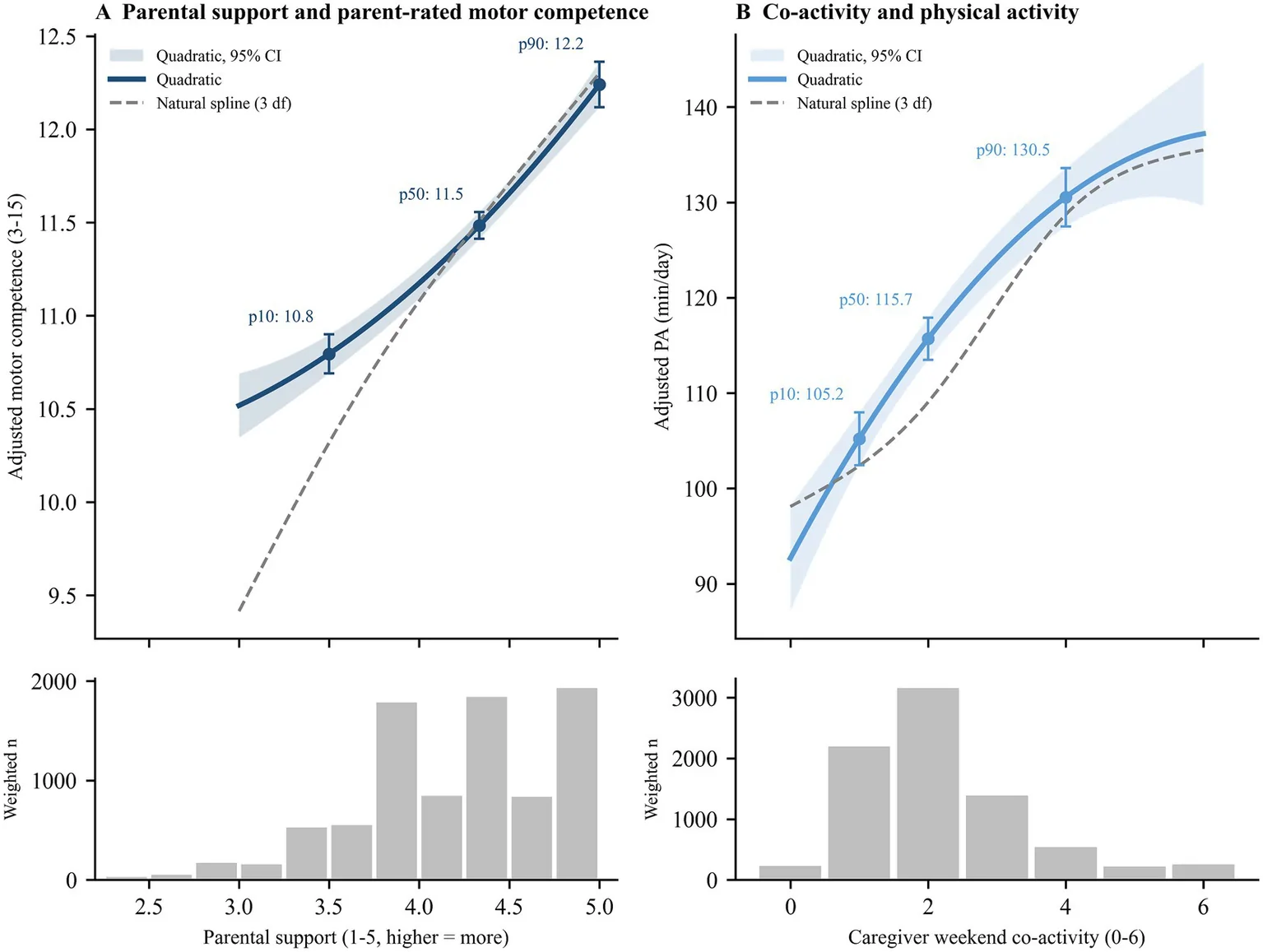
Adjusted curves for the two associations that departed from linearity. Survey-weighted regression of the outcome on the predictor, its square, and the five covariates (child age, sex, urban–rural residence, income, parental education), with design-based 95% confidence bands (shaded); dashed lines show a natural cubic spline (3 df) fitted as a check on the quadratic form. Points with error bars are adjusted predictions at the 10th, 50th, and 90th percentiles of the predictor. Lower panels show the weighted distribution of the predictor. **(A)** Parental support and parent-rated motor competence (*N* = 8,824); **(B)** caregiver weekend co-activity and physical activity (*N* = 8,059). Neither association reverses direction within the observed range.

### 3.3 Behavioral decomposition of the support–PA association

The association of parental support with each outcome was decomposed through the four measured parental behaviors in the main analytic samples (Table 3; Figure 3); all quantities are per one scale point of support. For PA, the total association was 13.8 min/day (bootstrap 95% CI 10.5–16.4), of which 5.5 min/day (4.4–6.6), or 40 percent (31–53), was statistically accounted for by the behaviors, and 8.3 min/day (5.3–10.9) was not. The estimated components were 3.11 min/day (2.36–3.79) for home sport equipment, 1.71 (1.18–2.28) for co-activity, and 0.86 (0.05–1.57) for maternal exercise; the paternal-exercise component was close to zero (−0.18, −0.83 to 0.47). For motor competence, the total association was 0.88 points (0.78–0.99), of which 0.15 points (0.11–0.18), or 17 percent (12–21), was accounted for by the behaviors, mainly home equipment (0.07, 0.05–0.09) with smaller components for paternal exercise (0.05, 0.02–0.08) and maternal exercise (0.03, 0.00–0.06) and none for co-activity (0.00, −0.01 to 0.01); the remaining 0.73 points (0.63–0.84) was not accounted for by the measured behaviors. With the four-item reduced-overlap support score, the pattern was the same, with the share of the PA association statistically accounted for by behaviors at 32 percent (24–41) and the motor share at 20 percent (16–26; Supplementary Table S12).

**Table 3:** Statistical decomposition of the association of parental support with each outcome through four parental behaviors.

Quantity	PA (min/day): estimate (bootstrap 95% CI)	Parent-rated motor competence (points): estimate (bootstrap 95% CI)
Total association (c)	+13.78 (+10.55, +16.35)	+0.883 (+0.782, +0.988)
Direct association (c′)	+8.29 (+5.28, +10.95)	+0.735 (+0.633, +0.836)
Total indirect (sum of *a* × *b*)	+5.49 (+4.36, +6.57)	+0.148 (+0.110, +0.185)
Indirect via home sport equipment	+3.11 (+2.36, +3.79)	+0.068 (+0.049, +0.088)
Indirect via caregiver co-activity	+1.71 (+1.18, +2.28)	+0.002 (−0.009, +0.015)
Indirect via mother’s exercise	+0.86 (+0.05, +1.57)	+0.031 (+0.000, +0.059)
Indirect via father’s exercise	−0.18 (−0.83, +0.47)	+0.046 (+0.020, +0.075)
a: support to home sport equipment	+0.56 (+0.47, +0.63)	+0.546 (+0.466, +0.622)
a: support to co-activity	+0.28 (+0.21, +0.34)	+0.273 (+0.210, +0.332)
a: support to mother’s exercise	+0.58 (+0.51, +0.63)	+0.595 (+0.540, +0.652)
a: support to father’s exercise	+0.56 (+0.49, +0.62)	+0.571 (+0.508, +0.630)
b: home sports equipment to outcome	+5.59 (+4.62, +6.57)	+0.125 (+0.094, +0.156)
b: co-activity to outcome	+6.10 (+4.85, +7.32)	+0.009 (−0.034, +0.051)
b: mother exercise to outcome	+1.49 (+0.09, +2.74)	+0.053 (+0.001, +0.098)
b: father exercise to outcome	−0.32 (−1.46, +0.86)	+0.081 (+0.036, +0.130)
Indirect share of total (%)	40 (31, 53)	17 (12, 21)

Product-of-coefficients decomposition in the main analytic samples (PA: *N* = 8,059; motor competence: *N* = 8,824), adjusted for child age, sex, urban–rural residence, household income, and parental education; all estimates per one scale point of parental support (SD of support = 0.56 in the PA sample and 0.56 in the motor sample; multiply by the SD to obtain per-SD values). Total indirect equals c minus c’ exactly in these linear models. 95% confidence intervals from 1,000 stratified cluster bootstrap resamples (county units resampled within province strata, weight retained). The decomposition is a cross-sectional statistical partition and does not establish mediation.

**Figure 3:**
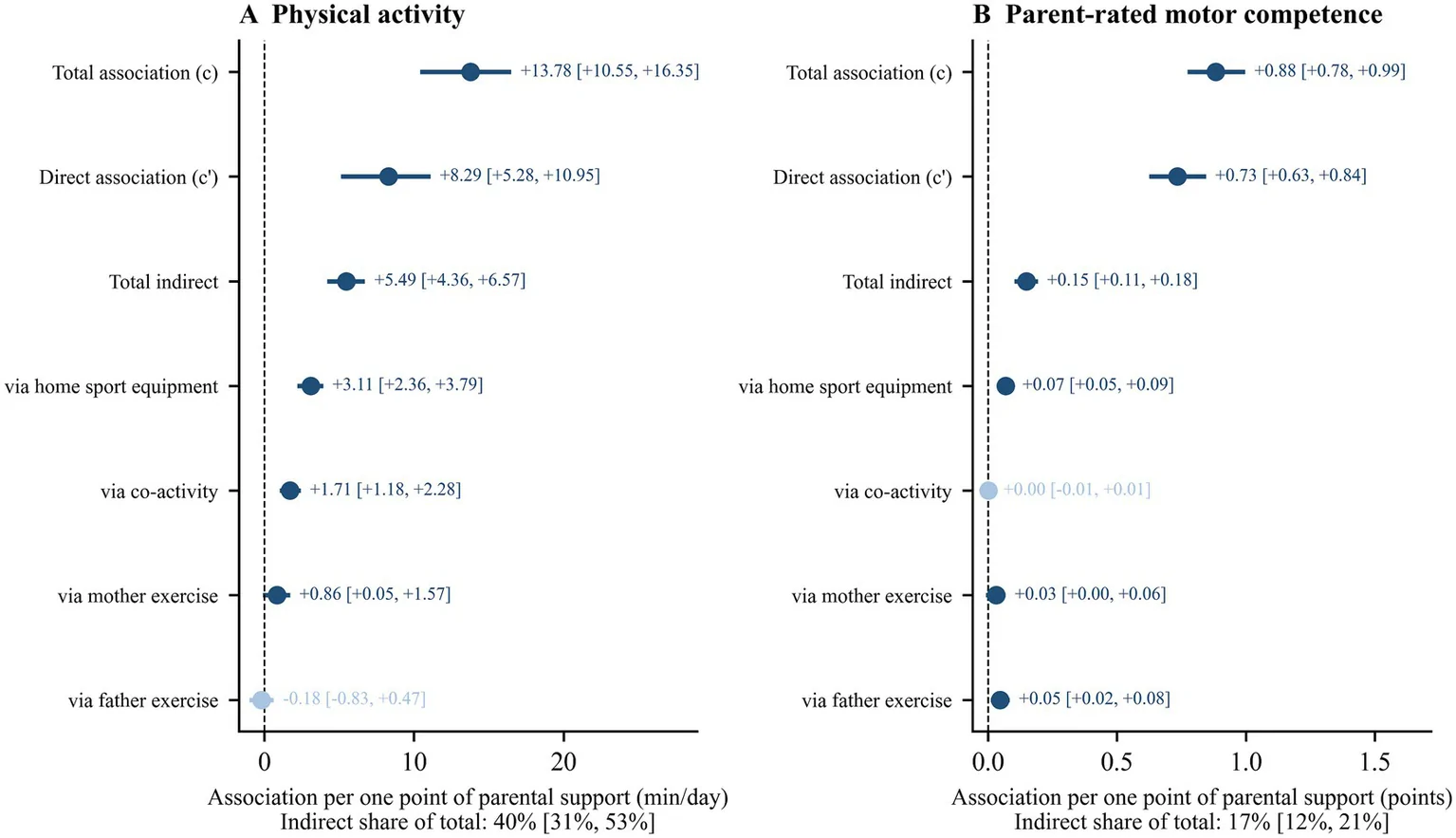
Statistical decomposition of the association of parental support with each outcome through four parental behaviors. Estimates per one scale point of parental support with 95% percentile bootstrap confidence intervals from 1,000 stratified cluster bootstrap resamples; filled markers indicate intervals that exclude zero. **(A)** Physical activity (min/day; *N* = 8,059); **(B)** parent-rated motor competence (points; *N* = 8,824). The total association (c) equals the direct association (c′) plus the sum of the four indirect components; the indirect share and its interval are given in each panel title. The decomposition is a cross-sectional statistical partition and does not establish mediation.

### 3.4 Exploratory moderation

A total of 12 interactions of support with child and household characteristics were tested (Supplementary Table S13). For PA, the support–PA association was stronger in households with higher parental education (interaction 2.2 min/day per SD of support per SD of education, 95% CI 0.9–3.6, *p* = 0.001; Benjamini–Hochberg-adjusted *p* = 0.013) and, at the nominal level only, in urban households (*p* = 0.037) and higher-income households (*p* = 0.036; both Benjamini–Hochberg-adjusted *p* = 0.15); the interaction with child sociability was not significant (*p* = 0.40). None of the six interactions for motor competence reached significance (all *p* ≥ 0.09). Because none of these tests were pre-registered, the education interaction is reported as exploratory.

### 3.5 Sensitivity

The positive association of parental screen time with child PA persisted after adjustment for home equipment and co-activity (father 3.7 min/day per SD, *p* < 0.001; mother 2.4 min/day per SD, *p* = 0.015), and the maternal coefficient attenuated under multiple imputation while its interval continued to exclude zero (1.8 min/day, 95% CI 0.18–3.52; Supplementary Table S10). Assigning 90 or 120 min instead of 75 to the open-ended activity category rescaled all PA coefficients upward without changing the signs or inference of the reported associations (Supplementary Table S7). Treating the internally standardized BMI index as a secondary outcome (*n* = 7,152; Supplementary Table S3), parental support (−0.03 SD per point, 95% CI −0.09 to 0.03) and paternal obesity (−0.01, −0.08 to 0.05) were not associated with the index; maternal obesity showed a small positive estimate for which neither the unadjusted nor the Benjamini–Hochberg-adjusted *p* value was below 0.05 (0.078 SD, 95% CI −0.003 to 0.158, *p* = 0.059), and paternal exercise frequency showed a small positive coefficient (0.05 SD per category, 95% CI 0.03–0.07, *p* < 0.001) that requires confirmation with measured anthropometry.

## 4 Discussion

### 4.1 Principal findings

The family associations differed between caregiver-reported physical activity and parent-rated motor competence. Parental support was positively associated with parent-rated motor competence, and the majority of this association was not statistically accounted for by the measured parental behaviors. Removing the enacted-support items attenuated the coefficient; the contrasts with child sociability and home equipment then no longer excluded zero. For PA, child sociability, home sport equipment, co-activity, and parental support were positively associated with activity volume. The contrasts between sociability and equipment and between co-activity and support did not exclude zero. Parental obesity was not associated with either outcome. These comparisons are conditional on the measures and covariates used.

### 4.2 Interpreting the differences between outcomes

The associations with reported activity volume concern the child’s disposition, the home material environment, and caregiver participation, domains considered in previous studies on children’s activity and motor skills (Barnett et al., 2013; Dowda et al., 2011; Pan et al., 2025a, 2025b; Patnode et al., 2010). Parent-rated motor competence captures a caregiver’s assessment of the child’s abilities relative to peers. Its association with support may reflect both differences between family environments and differences in caregivers’ perceptions. These possibilities cannot be separated using the present reports. Studies on family correlates and motor-skill interventions provide a basis for examining them prospectively (Cools et al., 2011; Logan et al., 2012). Social cognitive and bioecological accounts offer theoretical contexts for such studies (Bandura, 1986; Bronfenbrenner and Morris, 2006). The observed associations do not identify a process through which supportive caregiving changes children’s competence.

### 4.3 Decomposition of the associations

The decomposition describes the portion of the support association statistically accounted for by the measured behavioral variables. For PA, this portion involved home equipment and joint participation; for parent-rated motor competence, the majority of the support association remained after adjustment for these variables. The reduced-overlap analysis gave a smaller share for PA, including a smaller co-activity component than that obtained with the full scale. This sensitivity is consistent with partial overlap in what the support and co-activity items measure. These statistical components should not be interpreted as mediation.

### 4.4 Parental weight status

Familial aggregation of weight is well documented in older samples, but caregiver-reported parental obesity was not associated with the child’s activity or parent-rated competence. For the BMI index, paternal obesity was unrelated, and the maternal estimate was small and did not reach statistical significance (Supplementary Table S3). Within these measures and this precision, framing that treats parental body mass as a proxy for young-child activity or competence receives no support; the inference is bounded by the yes/no self-report of parental obesity and does not extend to adiposity outcomes measured objectively.

### 4.5 Findings that warrant caution

The sociability-PA association requires particular care because a caregiver who perceives a child as active may also report that child as more sociable. The positive association between parental screen time and child PA persisted after adjustment. Unmeasured household characteristics and reporting patterns are possible explanations; this finding provides no evidence that parental screen exposure benefits child activity. The exploratory interaction indicated a stronger support–PA association in households with higher parental education. Prospective replication would be needed before interpreting this difference as a difference in how children respond to support.

### 4.6 Strengths and limitations

The design is cross-sectional, so all estimates are associations, and causal direction is not identified; the behavioral decomposition is a statistical partition, not a test of mechanism. Every exposure and outcome was reported by the same caregiver. Shared-rater and common-method bias may therefore contribute to the associations, particularly between reported support and parent-rated motor competence. A caregiver who perceives an active child may also rate that child as more sociable or more supported. Adjustment for measured covariates and the reduced-overlap analysis do not remove this common reporting source. The support scale combines endorsement of activity with enacted support, and some items describe behavior of the kind that the co-activity measure also captures, so the exposure and a variable in the decomposition share measurement; Supplementary Table S12 shows how much the findings depend on those items. Motor competence was assessed using caregiver ratings, without an objective motor test. Daily PA was built from ordered duration categories with an assumed value for the open-ended category; the results were insensitive to that value, but category-based minutes cannot resolve within-category variation. Parental obesity was a yes/no self-report, which could attenuate obesity associations. Missing responses excluded part of the sample from the main models, with excluded children coming from less advantaged and less supportive households; although multiple imputation and inverse-probability weighting reproduced the estimates on which the conclusions rest, weights and imputation models cannot correct for selection on unmeasured factors. Pandemic conditions had reshaped household routines when the survey was fielded, although this context was shared across the sample. Finally, many associations were examined, and the exploratory interaction that remained significant after correction for multiple testing should be replicated before it is relied upon.

## 5 Conclusion

Parental support, child sociability, home sport equipment, and caregiver co-activity were associated with preschool children’s reported activity and parent-rated motor competence in patterns that differed by outcome and by the support measure used. Reported parental obesity was not associated with either outcome. The findings support examining these family and child characteristics together in longitudinal studies with independently assessed activity and motor skills.

## Data Availability

The participant-level survey records are curated by the China Institute of Sport Science and are not publicly available. Access requires approval from the General Administration of Sport of China, which owns the data; requests should be directed to the corresponding author, who will forward them for that approval. A variable dictionary that documents the source item, coding, and range of every analysis variable is provided as Supplementary Table S8, and the analysis code (R) is provided as Supplementary Data S1.

## Footnotes

1 Pan, X., Gao, Y., Jiang, L., Li, M., Suzuki, K., and Zhang, Y. (under review). Development, Validation, and Population Surveillance Application of a Brief Parental Support Scale for Preschooler Physical Activity in Chinese Families. Manuscript under Review.
