# Supplementary material for: Family correlates of physical activity and parent-rated motor competence in preschool children from a socioecological perspective: a cross-sectional analysis of 10,967 children in China
Source: Front Psychol. 2026 Oct 21;17:1898292. doi: 10.3389/fpsyg.2026.1898292 (PMC13730881; doi:10.3389/fpsyg.2026.1898292)
Supplement: Supplementary file 1 [file Supplementary_file_1.docx]

**Supplementary Material**

*Family correlates of physical activity and motor competence in preschool children from a socio-ecological perspective: a cross-sectional analysis of 10,967 children in China*

**Supplementary Table S1 | Physical activity items: wording, category-to-minute mapping, and weighted response distribution**

| **Item** | **Day type and segment** | **Response category** | **Assigned minutes** | **n (unweighted)** | **Weighted %** |
| --- | --- | --- | --- | --- | --- |
| B1_A | Weekday, total activity in and out of kindergarten; waking to 12:00 | No activity | 0 | 558 | 4.9 |
| B1_A | Weekday, total activity in and out of kindergarten; waking to 12:00 | 1-15 min | 8 | 1276 | 11.9 |
| B1_A | Weekday, total activity in and out of kindergarten; waking to 12:00 | 16-30 min | 23 | 3509 | 32.4 |
| B1_A | Weekday, total activity in and out of kindergarten; waking to 12:00 | 31-60 min | 45 | 2637 | 23.9 |
| B1_A | Weekday, total activity in and out of kindergarten; waking to 12:00 | >60 min | 75 (90 and 120 in sensitivity) | 2114 | 19.0 |
| B1_A | Weekday, total activity in and out of kindergarten; waking to 12:00 | Cannot say | missing | 867 | 7.9 |
| B1_B | Weekday, total activity in and out of kindergarten; 12:00 to 18:00 | No activity | 0 | 609 | 5.5 |
| B1_B | Weekday, total activity in and out of kindergarten; 12:00 to 18:00 | 1-15 min | 8 | 1013 | 9.6 |
| B1_B | Weekday, total activity in and out of kindergarten; 12:00 to 18:00 | 16-30 min | 23 | 3190 | 29.1 |
| B1_B | Weekday, total activity in and out of kindergarten; 12:00 to 18:00 | 31-60 min | 45 | 2847 | 25.9 |
| B1_B | Weekday, total activity in and out of kindergarten; 12:00 to 18:00 | >60 min | 75 (90 and 120 in sensitivity) | 2425 | 21.8 |
| B1_B | Weekday, total activity in and out of kindergarten; 12:00 to 18:00 | Cannot say | missing | 877 | 8.2 |
| B1_C | Weekday, total activity in and out of kindergarten; 18:00 to bedtime | No activity | 0 | 1321 | 11.7 |
| B1_C | Weekday, total activity in and out of kindergarten; 18:00 to bedtime | 1-15 min | 8 | 1332 | 12.4 |
| B1_C | Weekday, total activity in and out of kindergarten; 18:00 to bedtime | 16-30 min | 23 | 3061 | 28.3 |
| B1_C | Weekday, total activity in and out of kindergarten; 18:00 to bedtime | 31-60 min | 45 | 2379 | 21.4 |
| B1_C | Weekday, total activity in and out of kindergarten; 18:00 to bedtime | >60 min | 75 (90 and 120 in sensitivity) | 2355 | 21.5 |
| B1_C | Weekday, total activity in and out of kindergarten; 18:00 to bedtime | Cannot say | missing | 515 | 4.6 |
| B2_A | Weekend, out-of-home activity; waking to 12:00 | 1-15 min | 8 | 589 | 5.3 |
| B2_A | Weekend, out-of-home activity; waking to 12:00 | 16-30 min | 23 | 2063 | 19.1 |
| B2_A | Weekend, out-of-home activity; waking to 12:00 | 31-60 min | 45 | 2870 | 25.6 |
| B2_A | Weekend, out-of-home activity; waking to 12:00 | >60 min | 75 (90 and 120 in sensitivity) | 4102 | 38.1 |
| B2_A | Weekend, out-of-home activity; waking to 12:00 | No out-of-home activity | 0 | 1343 | 11.9 |
| B2_B | Weekend, out-of-home activity; 12:00 to 18:00 | 1-15 min | 8 | 518 | 4.7 |
| B2_B | Weekend, out-of-home activity; 12:00 to 18:00 | 16-30 min | 23 | 2086 | 19.3 |
| B2_B | Weekend, out-of-home activity; 12:00 to 18:00 | 31-60 min | 45 | 2988 | 27.0 |
| B2_B | Weekend, out-of-home activity; 12:00 to 18:00 | >60 min | 75 (90 and 120 in sensitivity) | 4156 | 37.9 |
| B2_B | Weekend, out-of-home activity; 12:00 to 18:00 | No out-of-home activity | 0 | 1219 | 11.0 |
| B2_C | Weekend, out-of-home activity; 18:00 to bedtime | 1-15 min | 8 | 922 | 8.4 |
| B2_C | Weekend, out-of-home activity; 18:00 to bedtime | 16-30 min | 23 | 2608 | 24.4 |
| B2_C | Weekend, out-of-home activity; 18:00 to bedtime | 31-60 min | 45 | 2730 | 25.0 |
| B2_C | Weekend, out-of-home activity; 18:00 to bedtime | >60 min | 75 (90 and 120 in sensitivity) | 2784 | 25.4 |
| B2_C | Weekend, out-of-home activity; 18:00 to bedtime | No out-of-home activity | 0 | 1921 | 16.8 |

*Weekday stem (B1): 'On school days (Monday to Friday), what is the average duration of your child's physical activity (kindergarten and outside kindergarten combined)?' Weekend stem (B2): 'On rest days (Saturday and Sunday), how much time does your child spend on out-of-home physical activity (including classes whose main content is physical activity, such as dance or football, but not calligraphy or piano)?' Daily average = (sum of three weekday segments x 5 + sum of three weekend segments x 2) / 7; possible range 0 to 225 min/day with the 75-minute assignment. Weekday and weekend items differ in what they count (all activity versus out-of-home activity), so the combined score is a caregiver-reported activity index rather than a measured duration.*

**Supplementary Table S2 | Adjusted predictions with 95% confidence intervals at the 10th, 50th, and 90th percentiles of the predictor for the two nonlinear associations**

| **Association** | **Percentile** | **Predictor value** | **Adjusted outcome (95% CI)** | **Quadratic term b** | **Quadratic term p** | **Spline monotone in range** | **N** |
| --- | --- | --- | --- | --- | --- | --- | --- |
| Parental support to motor competence | p10 | 3.50 | 10.80 (10.69, 10.90) | +0.205 | <0.001 | yes | 8824 |
| Parental support to motor competence | p50 | 4.33 | 11.48 (11.41, 11.56) | +0.205 | <0.001 | yes | 8824 |
| Parental support to motor competence | p90 | 5.00 | 12.24 (12.12, 12.36) | +0.205 | <0.001 | yes | 8824 |
| Co-activity to PA | p10 | 1.00 | 105.21 (102.44, 107.98) | -1.023 | 0.004 | yes | 8059 |
| Co-activity to PA | p50 | 2.00 | 115.70 (113.48, 117.91) | -1.023 | 0.004 | yes | 8059 |
| Co-activity to PA | p90 | 4.00 | 130.54 (127.47, 133.61) | -1.023 | 0.004 | yes | 8059 |

*Survey-weighted regression of the outcome on the predictor, its square, and the five covariates (child age, sex, urban-rural residence, income, parental education), design-based standard errors; predictions at weighted percentiles of the predictor with covariates at their weighted means. A natural cubic spline (3 df) was fitted as a check on the quadratic form; 'monotone' indicates that the spline prediction did not reverse direction across the 2nd to 98th percentile range. Curves with confidence bands and the weighted predictor distribution are shown in Figure 2.*

**Supplementary Table S3 | Secondary outcome: parental support, obesity, and exercise frequency and an internally standardized child BMI index**

| **Predictor** | **b (95% CI), SD units of the BMI index** | **p** | **p (BH-adjusted, 5 tests)** |
| --- | --- | --- | --- |
| Parental support (per point) | -0.033 (-0.092, +0.026) | 0.270 | 0.450 |
| Father obese | -0.014 (-0.079, +0.050) | 0.658 | 0.658 |
| Mother obese | +0.078 (-0.003, +0.158) | 0.059 | 0.149 |
| Father exercise frequency (per category) | +0.050 (+0.026, +0.073) | <0.001 | <0.001 |
| Mother exercise frequency (per category) | -0.007 (-0.030, +0.016) | 0.534 | 0.658 |

*N = 7,152 children with caregiver-reported height and weight. The BMI index is the residual of BMI on age in months and sex, standardized within the sample (mean 0, SD 1); it is not a growth-reference z-score. Survey-weighted regression adjusted for child age, sex, urban-rural residence, income, and parental education; design-based standard errors. Maternal obesity showed a small positive estimate, but neither the unadjusted nor the Benjamini-Hochberg-adjusted p value was below 0.05; paternal exercise frequency showed a small positive coefficient that persists after correction and warrants confirmation with measured anthropometry.*

**Supplementary Table S4 | Item content of the six-item parental support scale**

| Item | Content | Type |
| --- | --- | --- |
| 1 | Encouraging outdoor play in good weather | Encouragement |
| 2 | Parents setting an example in physical activity | Belief about role modeling |
| 3 | Importance of sustained physical activity for young children | Belief about the value of activity |
| 4 | Encouraging enrollment in organized sport or exercise classes | Encouragement |
| 5 | Taking the child to cultural and sport outings | Enacted support (behavior) |
| 6 | Active play with the child at home | Enacted support (behavior) |
| (not used) | Priority of academic study over exercise | Academic priority; not in the index |

*Five-point agreement scale (completely true to completely untrue), reverse-coded and averaged so that higher values indicate more support. Cronbach alpha = 0.76 for the six-item score and 0.74 for the four-item reduced-overlap score (items 1 to 4). The scale measures parental support for the child's activity rather than attitudes alone: items 5 and 6 describe enacted support and overlap with the caregiver co-activity measure, and items 1 and 4 describe encouragement, which refers to what the caregiver does as well as to what the caregiver endorses. The four-item score removes the overlap with co-activity but is not a pure attitude measure, and is used in the sensitivity analyses of Supplementary Table S12.*

**Supplementary Table S5 | Variable-specific missingness in the full sample (N = 10,967) and among children with an observed outcome**

| **Variable** | **Missing, n** | **Missing, %** | **Reason** | **Missing among PA-observed (n)** | **Missing among motor-observed (n)** |
| --- | --- | --- | --- | --- | --- |
| Daily PA | 1302 | 11.87 | 'cannot say' in at least one weekday segment | - | - |
| Motor competence | 8 | 0.07 | item not answered | - | - |
| Parental support | 14 | 0.13 | item not answered | 12 | 10 |
| Persistence | 1 | 0.01 | item not answered | 1 | 1 |
| Father exercise | 791 | 7.21 | 'unclear or not applicable' | 557 | 791 |
| Mother exercise | 596 | 5.43 | 'unclear or not applicable' | 402 | 596 |
| Father screen time | 1080 | 9.85 | 'cannot say' | 784 | 1080 |
| Mother screen time | 954 | 8.70 | 'cannot say' | 675 | 954 |
| Co-activity | 0 | 0.00 | - | 0 | 0 |
| Home sport equipment | 0 | 0.00 | - | 0 | 0 |
| Neighborhood environment | 10 | 0.09 | item not answered | 9 | 8 |
| Child sociability | 28 | 0.26 | item not answered | 24 | 24 |
| Household income | 2 | 0.02 | item not answered | 2 | 2 |
| Parental education | 4 | 0.04 | item not answered | 4 | 4 |
| Child age | 166 | 1.51 | birth date missing or implausible (outside 24-96 months) | 144 | 166 |
| Father obese | 0 | 0.00 | - | 0 | 0 |
| Mother obese | 0 | 0.00 | - | 0 | 0 |
| Sex | 0 | 0.00 | - | 0 | 0 |
| Urban-rural | 2 | 0.02 | item not answered | 2 | 2 |
| Parenting orientation | 5 | 0.05 | item not answered | 5 | 5 |
| Perceived child weight | 1 | 0.01 | item not answered | 1 | 1 |

*Participant flow: 10,967 children; PA scored for 9,665; PA analytic sample (all predictors observed) 8,059; motor competence scored for 10,959; motor analytic sample 8,824; both outcomes and all predictors observed 8,055 (Supplementary Figure S1).*

**Supplementary Table S6 | Weighted characteristics of children included in and excluded from each analytic sample**

| **Variable** | **PA sample: included (n), mean** | **PA sample: excluded (n), mean** | **Standardized difference** | **Motor sample: included (n), mean** | **Motor sample: excluded (n), mean** | **Standardized difference** |
| --- | --- | --- | --- | --- | --- | --- |
| Child age, months | (8,059) 65.78 | (2,742) 65.72 | +0.00 | (8,824) 65.71 | (1,977) 66.00 | -0.02 |
| Boys, proportion | (8,059) 0.54 | (2,908) 0.55 | -0.02 | (8,824) 0.54 | (2,143) 0.55 | -0.02 |
| Urban, proportion | (8,059) 0.64 | (2,906) 0.55 | +0.18 | (8,824) 0.64 | (2,141) 0.52 | +0.23 |
| Household income (1-10) | (8,059) 3.81 | (2,906) 3.24 | +0.29 | (8,824) 3.78 | (2,141) 3.15 | +0.33 |
| Parental education (1-5) | (8,059) 3.08 | (2,904) 2.79 | +0.31 | (8,824) 3.07 | (2,139) 2.74 | +0.36 |
| Parental support (1-5) | (8,059) 4.27 | (2,894) 4.02 | +0.41 | (8,824) 4.25 | (2,129) 4.00 | +0.40 |
| Child sociability (1-5) | (8,059) 3.48 | (2,880) 3.47 | +0.01 | (8,824) 3.48 | (2,115) 3.45 | +0.04 |
| Home sport equipment (0-10) | (8,059) 1.84 | (2,908) 1.60 | +0.15 | (8,824) 1.82 | (2,143) 1.59 | +0.14 |
| Co-activity (0-6) | (8,059) 2.19 | (2,908) 1.97 | +0.16 | (8,824) 2.19 | (2,143) 1.90 | +0.21 |
| Motor competence (3-15) | (8,055) 11.48 | (2,904) 11.22 | +0.12 | (8,824) 11.47 | (2,135) 11.19 | +0.13 |
| Daily PA, min/day | (8,059) 115.84 | (1,606) 107.86 | +0.14 | (8,055) 115.84 | (1,610) 107.84 | +0.14 |

*Weighted means among children with the variable observed; standardized difference = (included minus excluded) / pooled SD. Positive values indicate higher values among included children.*

**Supplementary Table S7 | Sensitivity of the physical activity model to the minutes assigned to the open-ended category (more than 60 minutes)**

| **Predictor** | **75 min: b (95% CI), p** | **90 min: b (95% CI), p** | **120 min: b (95% CI), p** |
| --- | --- | --- | --- |
| Child sociability (per SD) | +8.31 (+6.70, +9.92), <0.001 | +10.01 (+8.06, +11.97), <0.001 | +13.41 (+10.73, +16.09), <0.001 |
| Home sport equipment (per SD) | +8.01 (+6.47, +9.55), <0.001 | +9.59 (+7.69, +11.50), <0.001 | +12.76 (+10.09, +15.43), <0.001 |
| Caregiver co-activity (per SD) | +5.90 (+4.40, +7.41), <0.001 | +7.20 (+5.34, +9.07), <0.001 | +9.80 (+7.18, +12.42), <0.001 |
| Parental support (per SD) | +4.45 (+2.80, +6.10), <0.001 | +5.55 (+3.55, +7.56), <0.001 | +7.75 (+5.00, +10.51), <0.001 |
| Father screen time (per SD) | +3.81 (+1.87, +5.75), <0.001 | +4.85 (+2.45, +7.25), <0.001 | +6.93 (+3.57, +10.29), <0.001 |
| Mother screen time (per SD) | +2.84 (+0.99, +4.70), 0.003 | +3.41 (+1.11, +5.71), 0.004 | +4.54 (+1.32, +7.76), 0.006 |
| Mother exercise frequency (per SD) | +2.08 (+0.43, +3.74), 0.014 | +2.36 (+0.36, +4.36), 0.021 | +2.91 (+0.18, +5.64), 0.037 |
| Child age (per SD) | -2.73 (-4.16, -1.30), <0.001 | -3.43 (-5.17, -1.69), <0.001 | -4.83 (-7.23, -2.42), <0.001 |
| Mother obese (vs not) | +3.08 (-1.06, +7.22), 0.145 | +4.14 (-0.96, +9.23), 0.111 | +6.25 (-0.81, +13.30), 0.082 |
| Father obese (vs not) | -0.18 (-3.28, +2.93), 0.911 | +0.05 (-3.73, +3.83), 0.981 | +0.49 (-4.71, +5.69), 0.853 |
| Weighted mean PA, min/day | 114.5 | 125.7 | 148.3 |
| Possible maximum, min/day | 225 | 270 | 360 |
| Model R-squared | 0.115 | 0.112 | 0.106 |

*Same fully adjusted design-based model as Table 2 (N = 8,059); selected predictors shown. Coefficients scale with the assigned value because the outcome range widens; the ordering and significance of predictors are unchanged.*

**Supplementary Table S8 | Variable dictionary: source item, coding, and range of every analysis variable**

| **Analysis variable** | **Source item(s)** | **Coding** | **Range in analysis** |
| --- | --- | --- | --- |
| Daily PA (min/day) | B1_A, B1_B, B1_C (weekday); B2_A, B2_B, B2_C (weekend) | Categories to minutes as in Table S1; (weekday sum x 5 + weekend sum x 2)/7 | 0-225 |
| Parent-rated motor competence | A1_A, A1_B, A1_C | Each 1 (very poor) to 5 (excellent) relative to peers; summed | 3-15 |
| Parental support (6-item) | C13_A, C13_B, C13_D, C13_E, C13_F, C13_G | Each 1 (completely true) to 5 (completely untrue); 6 minus mean, so higher = more support; all items required | 1-5 |
| Parental support (4-item reduced-overlap, sensitivity) | C13_A, C13_B, C13_D, C13_E | As above, omitting the two enacted-support items | 1-5 |
| Parenting orientation | C10 | 1 preference-led (reference), 2 weakness-targeted, 3 knowledge-based; two indicators | categorical |
| Persistence when child resists | C11 | 1 let the child stop, 2 continue a while then decide, 3 insist on continuing; linear | 1-3 |
| Perceived child weight | C9 | 1 very fat, 2 rather fat (heavier); 3 about right (reference); 4 rather thin, 5 very thin (thinner); two indicators | categorical |
| Father / mother exercise frequency | C3 / C4 | 1 more than 3 times/week to 5 never; recoded 6 minus value so higher = more frequent; 6 (unclear or not applicable) = missing | 1-5 |
| Father / mother weekend screen time | C6 / C7 | 1 under 1 h to 5 4 h or more per day; 6 (cannot say) = missing | 1-5 |
| Caregiver weekend co-activity | C12 | 1 under 2 h, 2 2-4 h, 3 4-6 h, 4 6-8 h, 5 8-12 h, 6 12 h or more, 7 no time; 7 recoded to 0; linear | 0-6 |
| Father / mother obese | C14 / C15 | 1 yes = 1, 2 no = 0 | 0/1 |
| Home sport equipment | C8 (multiple choice) | Count of sport-related options selected: 3 (ball games, small), 4 basketball, 5 tennis, 6 volleyball, 8 football, 9 table tennis, 10 badminton, 14 bicycle, 15 scooter, 16 roller skates/skateboard | 0-10 |
| Neighborhood sport environment | D1_A to D1_E | Each 1 (completely true) to 5 (completely untrue); 6 minus mean, higher = better | 1-5 |
| Child sociability | C1_A (rather quiet), C1_B (dislikes company), C1_D (prefers to be alone) | Each 1 (completely true) to 5 (completely untrue); mean, so higher = more sociable | 1-5 |
| Child age (months) | F2_Y, F2_M (birth year, month) | (2020 minus year) x 12 + (10 minus month); values outside 24-96 set to missing | 24-96 |
| Sex | sex | 1 boy = 1, 2 girl = 0 | 0/1 |
| Urban-rural residence | Urban-rural classification of the sampled residential or village committee | urban = 1, rural = 0; 2 records carry no classification and are treated as missing | 0/1 |
| Household income | F12 | Ten ordered annual income bands (1 lowest to 10 highest); linear | 1-10 |
| Highest parental education | F11_A, F11_B | Maximum of father and mother, 1 primary or below to 5 postgraduate; linear | 1-5 |
| BMI index (secondary) | F3_A (height, cm), F3_B (weight, kg) | BMI outside 8-35 set to missing; residual on age and sex, standardized | SD units |
| Survey weight | W1 | Constructed for the survey; constant within province x urban-rural x sex cells; mean 1 | - |
| Sampling unit / stratum | County code / province code | 470 county-level units represented in this file, nested in 31 provinces (486 units in the national design); county labels recorded as free text were mapped to a single identifier per county | - |

*Item codes refer to the 2020 China National Fitness Activity Status Survey questionnaire for children aged 3 to 6 years (caregiver-completed). The C8 option numbers listed are those counted as sport equipment; the remaining options are non-sport toys and are not counted.*

**Supplementary Table S9 | Full main-model coefficients per natural unit, variance inflation factors, and province-clustered small-sample inference**

**Physical activity (min/day)**

| **Predictor** | **b per SD (design-based 95% CI)** | **b per natural unit** | **SD of predictor** | **VIF** | **p, design-based (county PSU)** | **p, province-clustered CR2 (Satterthwaite df)** |
| --- | --- | --- | --- | --- | --- | --- |
| Parental support (per SD) | +4.45 (+2.80, +6.10) | +8.013 | 0.56 | 1.34 | <0.001 | <0.001 (13.8) |
| Persistence when child resists (per SD) | -0.32 (-1.62, +0.97) | -0.544 | 0.60 | 1.04 | 0.624 | 0.656 (12.4) |
| Father exercise frequency (per SD) | +0.45 (-1.09, +1.98) | +0.360 | 1.24 | 1.53 | 0.569 | 0.574 (13.8) |
| Mother exercise frequency (per SD) | +2.08 (+0.43, +3.74) | +1.675 | 1.24 | 1.52 | 0.014 | 0.107 (13.3) |
| Father screen time (per SD) | +3.81 (+1.87, +5.75) | +3.053 | 1.25 | 1.79 | <0.001 | 0.002 (12.9) |
| Mother screen time (per SD) | +2.84 (+0.99, +4.70) | +2.432 | 1.17 | 1.75 | 0.003 | <0.001 (13.6) |
| Caregiver co-activity (per SD) | +5.90 (+4.40, +7.41) | +4.737 | 1.25 | 1.13 | <0.001 | <0.001 (13.5) |
| Home sport equipment (per SD) | +8.01 (+6.47, +9.55) | +4.971 | 1.61 | 1.13 | <0.001 | <0.001 (12.5) |
| Neighborhood sport environment (per SD) | +0.41 (-1.25, +2.08) | +0.542 | 0.76 | 1.23 | 0.627 | 0.600 (13.3) |
| Child sociability (per SD) | +8.31 (+6.70, +9.92) | +10.538 | 0.79 | 1.06 | <0.001 | <0.001 (13.2) |
| Household income (per SD) | +1.01 (-0.61, +2.63) | +0.494 | 2.05 | 1.39 | 0.222 | 0.276 (13.7) |
| Highest parental education (per SD) | -1.39 (-3.25, +0.46) | -1.474 | 0.95 | 1.51 | 0.140 | 0.201 (12.1) |
| Child age (per SD) | -2.73 (-4.16, -1.30) | -0.195 | 14.00 | 1.04 | <0.001 | 0.002 (12.5) |
| Father obese (vs not) | -0.18 (-3.28, +2.93) | -0.176 | - | 1.07 | 0.911 | 0.919 (12.8) |
| Mother obese (vs not) | +3.08 (-1.06, +7.22) | +3.081 | - | 1.07 | 0.145 | 0.110 (12.5) |
| Boy (vs girl) | -0.95 (-3.47, +1.56) | -0.950 | - | 1.01 | 0.458 | 0.472 (12.9) |
| Urban (vs rural) | -0.57 (-3.77, +2.64) | -0.567 | - | 1.18 | 0.728 | 0.733 (12.2) |
| Parenting orientation: weakness-targeted (vs preference-led) | +2.28 (-1.61, +6.16) | +2.277 | - | 1.08 | 0.250 | 0.369 (12.6) |
| Parenting orientation: knowledge-based (vs preference-led) | +2.17 (-2.20, +6.54) | +2.169 | - | 1.08 | 0.330 | 0.445 (14.0) |
| Perceived child weight: heavier than right (vs about right) | -4.55 (-9.62, +0.53) | -4.547 | - | 1.05 | 0.079 | 0.263 (11.5) |
| Perceived child weight: thinner than right (vs about right) | -2.61 (-5.95, +0.73) | -2.611 | - | 1.04 | 0.125 | 0.261 (12.4) |

**Parent-rated motor competence (points)**

| **Predictor** | **b per SD (design-based 95% CI)** | **b per natural unit** | **SD of predictor** | **VIF** | **p, design-based (county PSU)** | **p, province-clustered CR2 (Satterthwaite df)** |
| --- | --- | --- | --- | --- | --- | --- |
| Parental support (per SD) | +0.318 (+0.258, +0.378) | +0.567 | 0.56 | 1.34 | <0.001 | <0.001 (13.8) |
| Persistence when child resists (per SD) | -0.012 (-0.062, +0.038) | -0.020 | 0.60 | 1.04 | 0.642 | 0.706 (12.4) |
| Father exercise frequency (per SD) | +0.077 (+0.013, +0.140) | +0.061 | 1.26 | 1.51 | 0.018 | 0.003 (13.5) |
| Mother exercise frequency (per SD) | +0.053 (-0.010, +0.117) | +0.042 | 1.27 | 1.51 | 0.098 | 0.124 (12.9) |
| Father screen time (per SD) | -0.092 (-0.165, -0.018) | -0.073 | 1.26 | 1.77 | 0.014 | 0.023 (13.0) |
| Mother screen time (per SD) | -0.046 (-0.112, +0.021) | -0.039 | 1.18 | 1.72 | 0.177 | 0.188 (13.6) |
| Caregiver co-activity (per SD) | +0.033 (-0.024, +0.091) | +0.026 | 1.26 | 1.13 | 0.258 | 0.264 (13.7) |
| Home sport equipment (per SD) | +0.177 (+0.126, +0.228) | +0.110 | 1.61 | 1.13 | <0.001 | <0.001 (12.7) |
| Neighborhood sport environment (per SD) | +0.163 (+0.109, +0.216) | +0.211 | 0.77 | 1.23 | <0.001 | <0.001 (13.6) |
| Child sociability (per SD) | +0.191 (+0.133, +0.249) | +0.242 | 0.79 | 1.06 | <0.001 | <0.001 (13.4) |
| Household income (per SD) | +0.062 (-0.001, +0.126) | +0.030 | 2.06 | 1.38 | 0.054 | 0.050 (13.7) |
| Highest parental education (per SD) | +0.030 (-0.036, +0.096) | +0.032 | 0.95 | 1.51 | 0.366 | 0.493 (12.1) |
| Child age (per SD) | -0.066 (-0.116, -0.016) | -0.005 | 14.04 | 1.04 | 0.010 | 0.037 (12.7) |
| Father obese (vs not) | +0.002 (-0.113, +0.117) | +0.002 | - | 1.07 | 0.970 | 0.970 (12.9) |
| Mother obese (vs not) | +0.021 (-0.129, +0.170) | +0.021 | - | 1.07 | 0.786 | 0.776 (12.4) |
| Boy (vs girl) | +0.041 (-0.049, +0.130) | +0.041 | - | 1.01 | 0.370 | 0.397 (13.0) |
| Urban (vs rural) | -0.118 (-0.235, -0.002) | -0.118 | - | 1.18 | 0.047 | 0.121 (12.2) |
| Parenting orientation: weakness-targeted (vs preference-led) | -0.265 (-0.387, -0.142) | -0.265 | - | 1.08 | <0.001 | <0.001 (12.7) |
| Parenting orientation: knowledge-based (vs preference-led) | +0.032 (-0.102, +0.166) | +0.032 | - | 1.08 | 0.640 | 0.615 (13.8) |
| Perceived child weight: heavier than right (vs about right) | -0.136 (-0.395, +0.123) | -0.136 | - | 1.05 | 0.303 | 0.292 (11.5) |
| Perceived child weight: thinner than right (vs about right) | -0.363 (-0.497, -0.230) | -0.363 | - | 1.04 | <0.001 | <0.001 (12.4) |

*Design-based inference uses county-level sampling units nested in province strata (Taylor linearization). The province-clustered column uses cluster-robust variance with the CR2 small-sample correction and Satterthwaite degrees of freedom (31 clusters). Point estimates are identical across columns.*

**Supplementary Table S10 | Missing-data sensitivity: complete-case, multiple imputation, and inverse-probability-weighted estimates**

**Physical activity (min/day)**

| **Predictor** | **Complete case: b (95% CI)** | **Multiple imputation: b (95% CI)** | **FMI** | **IPW, extended model: b (95% CI)** | **IPW, demographic model: b (95% CI)** |
| --- | --- | --- | --- | --- | --- |
| Child sociability (per SD) | +8.31 (+6.70, +9.92) | +8.51 (+7.10, +9.92) | 9 % | +8.29 (+6.62, +9.97) | +8.30 (+6.66, +9.94) |
| Home sport equipment (per SD) | +8.01 (+6.47, +9.55) | +8.12 (+6.67, +9.58) | 7 % | +8.12 (+6.53, +9.71) | +8.12 (+6.55, +9.69) |
| Caregiver co-activity (per SD) | +5.90 (+4.40, +7.41) | +6.52 (+5.11, +7.93) | 9 % | +5.91 (+4.39, +7.44) | +5.84 (+4.34, +7.35) |
| Perceived child weight: heavier than right (vs about right) | -4.55 (-9.62, +0.53) | -2.29 (-7.42, +2.84) | 14 % | -4.73 (-10.12, +0.67) | -4.29 (-9.47, +0.89) |
| Parental support (per SD) | +4.45 (+2.80, +6.10) | +4.51 (+2.93, +6.10) | 11 % | +4.54 (+2.88, +6.21) | +4.33 (+2.67, +6.00) |
| Father screen time (per SD) | +3.81 (+1.87, +5.75) | +4.22 (+2.39, +6.05) | 13 % | +3.88 (+1.94, +5.83) | +3.92 (+1.96, +5.89) |
| Mother obese (vs not) | +3.08 (-1.06, +7.22) | +2.62 (-1.38, +6.61) | 12 % | +3.47 (-0.76, +7.70) | +3.06 (-1.09, +7.21) |
| Mother screen time (per SD) | +2.84 (+0.99, +4.70) | +1.85 (+0.18, +3.52) | 15 % | +2.65 (+0.77, +4.53) | +2.87 (+0.99, +4.76) |
| Child age (per SD) | -2.73 (-4.16, -1.30) | -3.04 (-4.28, -1.81) | 6 % | -2.63 (-4.07, -1.19) | -2.69 (-4.13, -1.25) |
| Perceived child weight: thinner than right (vs about right) | -2.61 (-5.95, +0.73) | -0.56 (-4.02, +2.90) | 15 % | -2.69 (-6.15, +0.76) | -2.55 (-5.95, +0.85) |
| Parenting orientation: weakness-targeted (vs preference-led) | +2.28 (-1.61, +6.16) | +3.17 (-0.58, +6.92) | 7 % | +2.31 (-1.68, +6.29) | +2.26 (-1.68, +6.20) |
| Parenting orientation: knowledge-based (vs preference-led) | +2.17 (-2.20, +6.54) | +2.66 (-1.29, +6.61) | 8 % | +1.99 (-2.46, +6.43) | +1.91 (-2.49, +6.30) |
| Mother exercise frequency (per SD) | +2.08 (+0.43, +3.74) | +2.11 (+0.51, +3.71) | 14 % | +2.01 (+0.31, +3.70) | +1.94 (+0.28, +3.60) |
| Highest parental education (per SD) | -1.39 (-3.25, +0.46) | -1.48 (-3.13, +0.17) | 13 % | -1.72 (-3.59, +0.15) | -1.40 (-3.25, +0.46) |
| Household income (per SD) | +1.01 (-0.61, +2.63) | +1.02 (-0.53, +2.57) | 8 % | +1.31 (-0.37, +2.99) | +1.13 (-0.51, +2.77) |
| Boy (vs girl) | -0.95 (-3.47, +1.56) | -1.31 (-3.57, +0.95) | 9 % | -1.02 (-3.61, +1.57) | -0.95 (-3.51, +1.61) |
| Urban (vs rural) | -0.57 (-3.77, +2.64) | -1.56 (-4.58, +1.45) | 4 % | -0.52 (-3.72, +2.67) | -0.54 (-3.73, +2.66) |
| Father exercise frequency (per SD) | +0.45 (-1.09, +1.98) | +0.96 (-0.56, +2.47) | 19 % | +0.21 (-1.34, +1.76) | +0.32 (-1.23, +1.88) |
| Neighborhood sport environment (per SD) | +0.41 (-1.25, +2.08) | +0.60 (-0.91, +2.11) | 7 % | +0.39 (-1.30, +2.09) | +0.40 (-1.30, +2.09) |
| Persistence when child resists (per SD) | -0.32 (-1.62, +0.97) | -0.04 (-1.28, +1.21) | 6 % | -0.56 (-1.89, +0.76) | -0.40 (-1.72, +0.91) |
| Father obese (vs not) | -0.18 (-3.28, +2.93) | -0.48 (-3.41, +2.46) | 8 % | -0.64 (-3.85, +2.57) | -0.07 (-3.21, +3.07) |

**Parent-rated motor competence (points)**

| **Predictor** | **Complete case: b (95% CI)** | **Multiple imputation: b (95% CI)** | **FMI** | **IPW, extended model: b (95% CI)** | **IPW, demographic model: b (95% CI)** |
| --- | --- | --- | --- | --- | --- |
| Perceived child weight: thinner than right (vs about right) | -0.363 (-0.497, -0.230) | -0.361 (-0.485, -0.237) | 0 % | -0.360 (-0.496, -0.225) | -0.362 (-0.497, -0.228) |
| Parental support (per SD) | +0.318 (+0.258, +0.378) | +0.312 (+0.259, +0.365) | 1 % | +0.321 (+0.260, +0.382) | +0.323 (+0.263, +0.384) |
| Parenting orientation: weakness-targeted (vs preference-led) | -0.265 (-0.387, -0.142) | -0.257 (-0.369, -0.144) | 0 % | -0.258 (-0.383, -0.133) | -0.264 (-0.388, -0.140) |
| Child sociability (per SD) | +0.191 (+0.133, +0.249) | +0.197 (+0.142, +0.252) | 0 % | +0.193 (+0.135, +0.252) | +0.190 (+0.132, +0.249) |
| Home sport equipment (per SD) | +0.177 (+0.126, +0.228) | +0.161 (+0.113, +0.209) | 0 % | +0.177 (+0.125, +0.229) | +0.174 (+0.123, +0.225) |
| Neighborhood sport environment (per SD) | +0.163 (+0.109, +0.216) | +0.148 (+0.099, +0.196) | 0 % | +0.153 (+0.097, +0.208) | +0.162 (+0.108, +0.215) |
| Perceived child weight: heavier than right (vs about right) | -0.136 (-0.395, +0.123) | -0.081 (-0.309, +0.147) | 0 % | -0.123 (-0.376, +0.130) | -0.133 (-0.392, +0.125) |
| Urban (vs rural) | -0.118 (-0.235, -0.002) | -0.126 (-0.234, -0.019) | 0 % | -0.110 (-0.227, +0.008) | -0.120 (-0.237, -0.003) |
| Father screen time (per SD) | -0.092 (-0.165, -0.018) | -0.083 (-0.147, -0.018) | 9 % | -0.082 (-0.157, -0.008) | -0.089 (-0.163, -0.015) |
| Father exercise frequency (per SD) | +0.077 (+0.013, +0.140) | +0.094 (+0.035, +0.152) | 11 % | +0.080 (+0.016, +0.144) | +0.077 (+0.013, +0.141) |
| Child age (per SD) | -0.066 (-0.116, -0.016) | -0.070 (-0.115, -0.024) | 2 % | -0.064 (-0.116, -0.013) | -0.065 (-0.116, -0.015) |
| Household income (per SD) | +0.062 (-0.001, +0.126) | +0.085 (+0.029, +0.141) | 0 % | +0.063 (-0.002, +0.128) | +0.060 (-0.004, +0.124) |
| Mother exercise frequency (per SD) | +0.053 (-0.010, +0.117) | +0.049 (-0.007, +0.106) | 6 % | +0.047 (-0.018, +0.111) | +0.050 (-0.015, +0.114) |
| Mother screen time (per SD) | -0.046 (-0.112, +0.021) | -0.050 (-0.110, +0.010) | 10 % | -0.059 (-0.127, +0.008) | -0.047 (-0.114, +0.021) |
| Boy (vs girl) | +0.041 (-0.049, +0.130) | +0.031 (-0.049, +0.110) | 0 % | +0.044 (-0.047, +0.136) | +0.044 (-0.045, +0.133) |
| Caregiver co-activity (per SD) | +0.033 (-0.024, +0.091) | +0.024 (-0.028, +0.076) | 0 % | +0.030 (-0.028, +0.088) | +0.033 (-0.025, +0.091) |
| Parenting orientation: knowledge-based (vs preference-led) | +0.032 (-0.102, +0.166) | -0.038 (-0.160, +0.084) | 0 % | +0.010 (-0.125, +0.145) | +0.020 (-0.114, +0.155) |
| Highest parental education (per SD) | +0.030 (-0.036, +0.096) | +0.035 (-0.026, +0.096) | 0 % | +0.032 (-0.034, +0.098) | +0.031 (-0.035, +0.097) |
| Mother obese (vs not) | +0.021 (-0.129, +0.170) | +0.046 (-0.088, +0.180) | 0 % | +0.024 (-0.129, +0.176) | +0.018 (-0.132, +0.169) |
| Persistence when child resists (per SD) | -0.012 (-0.062, +0.038) | +0.008 (-0.039, +0.055) | 0 % | -0.013 (-0.064, +0.038) | -0.014 (-0.065, +0.036) |
| Father obese (vs not) | +0.002 (-0.113, +0.117) | -0.001 (-0.101, +0.100) | 0 % | +0.008 (-0.109, +0.126) | +0.002 (-0.114, +0.117) |

*Multiple imputation: 20 data sets by chained equations (predictive mean matching for numeric variables, polytomous regression for the two nominal variables), N = 10,967 in each; the imputation model contained both outcomes, all 19 predictors, the survey weight, province indicators, and the county-level (sampling-unit) means of the analysis variables, so that the stratification and the clustering of the design were represented in the imputation as well as in the analysis; the design-based model was fitted in each imputed data set and pooled by Rubin's rules; FMI = fraction of missing information. IPW: the survey weight was multiplied by the inverse of the predicted probability of being a complete case. The extended completeness model contains child age, sex, urban-rural residence, household income, parental education, every further predictor that was at least 99 percent complete (parental support, home sport equipment, co-activity, neighborhood environment, child sociability, persistence, parenting orientation, perceived child weight, and paternal and maternal obesity), and province indicators; its coefficients are given in Supplementary Table S15. The demographic model is the five-covariate model reported in the first revision and is retained for comparison.*

**Supplementary Table S11 | Relative importance (LMG share of R-squared) and pairwise contrasts between selected coefficients**

| **Predictor** | **PA: LMG share of decomposable R-squared (%)** | **Motor: LMG share of decomposable R-squared (%)** |
| --- | --- | --- |
| Parental support | 10.1 | 34.2 |
| Home sport equipment | 26.6 | 12.2 |
| Child sociability | 27.2 | 11.2 |
| Co-activity | 17.1 | 1.1 |
| Decomposable R-squared (total minus covariates) | 0.105 (total 0.115) | 0.095 (total 0.104) |

*Lindeman-Merenda-Gold decomposition of the weighted R-squared (averaged over all orderings of the 14 focal predictors, with the five covariates always included; computed from the weighted covariance matrix of the analytic sample). Shares are expressed as percentages of the R-squared assigned to the focal predictors. Selected predictors are shown.*

| **Outcome** | **Contrast (semi-standardized coefficients)** | **Difference (95% CI)** | **p** |
| --- | --- | --- | --- |
| PA | sociability minus home equipment | +0.31 (-1.98, +2.59) | 0.793 |
| PA | co-activity minus parental support | +1.45 (-0.86, +3.77) | 0.217 |
| Motor competence | parental support minus sociability | +0.127 (+0.039, +0.215) | 0.005 |
| Motor competence | parental support minus home equipment | +0.142 (+0.060, +0.224) | <0.001 |

*Linear contrasts between coefficients from the fully adjusted design-based models of Table 2.*

**Supplementary Table S12 | Sensitivity analysis with the four-item reduced-overlap support score (the two enacted-support items excluded)**

| **Quantity** | **Six-item score (main analysis)** | **Four-item score** |
| --- | --- | --- |
| PA: support coefficient per SD (95% CI) | +4.45 (+2.80, +6.10) | +4.48 (+2.91, +6.04) |
| PA: total association c (per point) | +13.78 (+10.55, +16.35) | +13.93 (+11.02, +16.80) |
| PA: direct association c' (per point) | +8.29 (+5.28, +10.95) | +9.52 (+6.79, +12.19) |
| PA: total indirect (per point) | +5.49 (+4.36, +6.57) | +4.41 (+3.40, +5.38) |
| PA: indirect via co-activity (per point) | +1.71 (+1.18, +2.28) | +0.98 (+0.52, +1.48) |
| PA: indirect share of total (%) | 40 (31, 53) | 32 (24, 41) |
| Motor: support coefficient per SD (95% CI) | +0.318 (+0.258, +0.378) | +0.225 (+0.166, +0.283) |
| Motor: total association c (per point) | +0.883 (+0.782, +0.988) | +0.723 (+0.616, +0.819) |
| Motor: direct association c' (per point) | +0.735 (+0.633, +0.836) | +0.576 (+0.475, +0.668) |
| Motor: total indirect (per point) | +0.148 (+0.110, +0.185) | +0.147 (+0.118, +0.183) |
| Motor: indirect via co-activity (per point) | +0.002 (-0.009, +0.015) | +0.004 (-0.003, +0.011) |
| Motor: indirect share of total (%) | 17 (12, 21) | 20 (16, 26) |

| **Outcome** | **Contrast with the four-item score** | **Difference (95% CI)** | **p** |
| --- | --- | --- | --- |
| PA | sociability minus home equipment | +0.14 (-2.14, +2.41) | 0.905 |
| PA | co-activity minus parental support | +1.67 (-0.45, +3.79) | 0.121 |
| Motor competence | parental support minus sociability | +0.036 (-0.053, +0.124) | 0.427 |
| Motor competence | parental support minus home equipment | +0.035 (-0.047, +0.117) | 0.399 |

*The four-item score omits the two enacted-support items (family outings, home co-activity), which overlap with the caregiver co-activity measure. Main models, pairwise contrasts, and decompositions were re-estimated exactly as in Tables 2 and 3. The support coefficient for parent-rated motor competence falls with the reduced score, and its contrasts with child sociability and home sport equipment no longer exclude zero; the indirect share of the support-PA association falls, which is what the part-whole overlap predicts.*

**Supplementary Table S13 | All tested interactions of parental support with child and household characteristics**

| **Outcome** | **Interaction** | **b (95% CI)** | **p** | **p (BH, 12 tests)** |
| --- | --- | --- | --- | --- |
| PA | support x sociable | +0.58 (-0.76, +1.93) | 0.396 | 0.475 |
| PA | support x male | +0.89 (-1.56, +3.35) | 0.475 | 0.518 |
| PA | support x urban | +3.28 (+0.20, +6.36) | 0.037 | 0.149 |
| PA | support x income | +1.60 (+0.10, +3.10) | 0.036 | 0.149 |
| PA | support x edu | +2.24 (+0.90, +3.58) | 0.001 | 0.013 |
| PA | support x age_m | -0.83 (-2.15, +0.49) | 0.217 | 0.434 |
| Motor competence | support x sociable | -0.005 (-0.063, +0.053) | 0.870 | 0.870 |
| Motor competence | support x male | -0.046 (-0.143, +0.051) | 0.352 | 0.475 |
| Motor competence | support x urban | -0.082 (-0.191, +0.027) | 0.139 | 0.333 |
| Motor competence | support x income | -0.024 (-0.079, +0.030) | 0.381 | 0.475 |
| Motor competence | support x edu | -0.025 (-0.077, +0.027) | 0.348 | 0.475 |
| Motor competence | support x age_m | -0.045 (-0.097, +0.007) | 0.093 | 0.279 |

*Each interaction term was added separately to the fully adjusted design-based model; support and continuous moderators are per SD. None of the interactions were pre-registered; all are exploratory.*

**Supplementary Table S14 | Sensitivity of the design-based inference to the stratum specification**

**Physical activity (min/day)**

| **Predictor** | **b** | **SE, province strata** | **p** | **SE, province x urban-rural strata** | **p** | **SE ratio** |
| --- | --- | --- | --- | --- | --- | --- |
| Parental support (per SD) | +4.45 | 0.84 | <0.001 | 0.85 | <0.001 | 1.013 |
| Persistence when child resists (per SD) | -0.32 | 0.66 | 0.624 | 0.67 | 0.627 | 1.009 |
| Father exercise frequency (per SD) | +0.45 | 0.78 | 0.569 | 0.78 | 0.568 | 0.997 |
| Mother exercise frequency (per SD) | +2.08 | 0.84 | 0.014 | 0.85 | 0.014 | 1.006 |
| Father screen time (per SD) | +3.81 | 0.99 | <0.001 | 0.97 | <0.001 | 0.988 |
| Mother screen time (per SD) | +2.84 | 0.94 | 0.003 | 0.93 | 0.002 | 0.984 |
| Caregiver co-activity (per SD) | +5.90 | 0.76 | <0.001 | 0.78 | <0.001 | 1.015 |
| Home sport equipment (per SD) | +8.01 | 0.78 | <0.001 | 0.77 | <0.001 | 0.988 |
| Neighborhood sport environment (per SD) | +0.41 | 0.85 | 0.627 | 0.86 | 0.632 | 1.014 |
| Child sociability (per SD) | +8.31 | 0.82 | <0.001 | 0.82 | <0.001 | 1.003 |
| Household income (per SD) | +1.01 | 0.83 | 0.222 | 0.83 | 0.222 | 1.001 |
| Highest parental education (per SD) | -1.39 | 0.94 | 0.140 | 0.95 | 0.142 | 1.006 |
| Child age (per SD) | -2.73 | 0.73 | <0.001 | 0.73 | <0.001 | 1.004 |
| Father obese (vs not) | -0.18 | 1.58 | 0.911 | 1.56 | 0.910 | 0.987 |
| Mother obese (vs not) | +3.08 | 2.11 | 0.145 | 2.11 | 0.145 | 1.001 |
| Boy (vs girl) | -0.95 | 1.28 | 0.458 | 1.26 | 0.450 | 0.983 |
| Urban (vs rural) | -0.57 | 1.63 | 0.728 | 1.63 | 0.728 | 0.998 |
| Parenting orientation: weakness-targeted (vs preference-led) | +2.28 | 1.98 | 0.250 | 1.96 | 0.245 | 0.990 |
| Parenting orientation: knowledge-based (vs preference-led) | +2.17 | 2.23 | 0.330 | 2.24 | 0.333 | 1.006 |
| Perceived child weight: heavier than right (vs about right) | -4.55 | 2.58 | 0.079 | 2.57 | 0.078 | 0.995 |
| Perceived child weight: thinner than right (vs about right) | -2.61 | 1.70 | 0.125 | 1.71 | 0.127 | 1.005 |

**Parent-rated motor competence (points)**

| **Predictor** | **b** | **SE, province strata** | **p** | **SE, province x urban-rural strata** | **p** | **SE ratio** |
| --- | --- | --- | --- | --- | --- | --- |
| Parental support (per SD) | +0.318 | 0.030 | <0.001 | 0.030 | <0.001 | 1.000 |
| Persistence when child resists (per SD) | -0.012 | 0.025 | 0.642 | 0.026 | 0.643 | 1.002 |
| Father exercise frequency (per SD) | +0.077 | 0.032 | 0.018 | 0.032 | 0.018 | 0.996 |
| Mother exercise frequency (per SD) | +0.053 | 0.032 | 0.098 | 0.032 | 0.092 | 0.983 |
| Father screen time (per SD) | -0.092 | 0.037 | 0.014 | 0.037 | 0.013 | 0.982 |
| Mother screen time (per SD) | -0.046 | 0.034 | 0.177 | 0.034 | 0.177 | 0.998 |
| Caregiver co-activity (per SD) | +0.033 | 0.029 | 0.258 | 0.029 | 0.259 | 1.002 |
| Home sport equipment (per SD) | +0.177 | 0.026 | <0.001 | 0.026 | <0.001 | 1.006 |
| Neighborhood sport environment (per SD) | +0.163 | 0.027 | <0.001 | 0.027 | <0.001 | 0.989 |
| Child sociability (per SD) | +0.191 | 0.029 | <0.001 | 0.029 | <0.001 | 1.001 |
| Household income (per SD) | +0.062 | 0.032 | 0.054 | 0.032 | 0.054 | 1.002 |
| Highest parental education (per SD) | +0.030 | 0.034 | 0.366 | 0.034 | 0.370 | 1.008 |
| Child age (per SD) | -0.066 | 0.025 | 0.010 | 0.026 | 0.010 | 1.004 |
| Father obese (vs not) | +0.002 | 0.058 | 0.970 | 0.058 | 0.970 | 0.995 |
| Mother obese (vs not) | +0.021 | 0.076 | 0.786 | 0.077 | 0.788 | 1.009 |
| Boy (vs girl) | +0.041 | 0.046 | 0.370 | 0.045 | 0.367 | 0.994 |
| Urban (vs rural) | -0.118 | 0.059 | 0.047 | 0.059 | 0.047 | 1.002 |
| Parenting orientation: weakness-targeted (vs preference-led) | -0.265 | 0.062 | <0.001 | 0.062 | <0.001 | 1.003 |
| Parenting orientation: knowledge-based (vs preference-led) | +0.032 | 0.068 | 0.640 | 0.068 | 0.638 | 0.995 |
| Perceived child weight: heavier than right (vs about right) | -0.136 | 0.132 | 0.303 | 0.131 | 0.301 | 0.997 |
| Perceived child weight: thinner than right (vs about right) | -0.363 | 0.068 | <0.001 | 0.069 | <0.001 | 1.016 |

*Point estimates are identical under the two specifications; only the variance estimator changes. Urban-rural classification is applied by the design at the second stage, to fix the number of urban and rural committees within a selected county, and is therefore an attribute of the sampled committee rather than of the county: 405 of the 470 county-level units contain both urban and rural respondents, so urban-rural classification does not partition the primary sampling units and cannot serve as a stratum above them. For this sensitivity analysis each county was assigned its majority classification, giving 59 province-by-urban-rural strata. Standard errors change by no more than a few percent and no p value crosses 0.05.*

**Supplementary Table S15 | Logistic model for complete-case inclusion used in the inverse-probability-weighted analysis**

| **Term** | **b** | **SE** | **p** |
| --- | --- | --- | --- |
| Intercept | -2.171 | 0.299 | <0.001 |
| Child age (months) | +0.003 | 0.002 | 0.042 |
| Boy | -0.015 | 0.047 | 0.740 |
| Urban | +0.072 | 0.052 | 0.169 |
| Household income | +0.070 | 0.015 | <0.001 |
| Parental education | +0.166 | 0.031 | <0.001 |
| Parental support | +0.489 | 0.044 | <0.001 |
| Home sport equipment | +0.035 | 0.016 | 0.027 |
| Co-activity | +0.025 | 0.019 | 0.188 |
| Neighborhood environment | +0.261 | 0.032 | <0.001 |
| Child sociability | -0.067 | 0.030 | 0.027 |
| Persistence | -0.062 | 0.038 | 0.102 |
| Parenting orientation: weakness-targeted | +0.301 | 0.061 | <0.001 |
| Parenting orientation: knowledge-based | +0.023 | 0.066 | 0.725 |
| Perceived weight: heavier | +0.069 | 0.092 | 0.451 |
| Perceived weight: thinner | -0.103 | 0.059 | 0.082 |
| Father obese | -0.026 | 0.056 | 0.648 |
| Mother obese | -0.111 | 0.067 | 0.097 |

*Outcome: inclusion in the physical activity analytic sample; N = 10,756 children with all model variables observed. Province indicators were included and are omitted from the table. The model contains every predictor that was at least 99 percent complete, including the three variables on which included and excluded children differ most (parental support, home sport equipment, co-activity; Supplementary Table S6). Inclusion could not be modeled on the variables that themselves drive exclusion (parental exercise frequency, parental screen time, weekday activity), so the inverse-probability-weighted estimates remain a sensitivity analysis under a missing-at-random assumption conditional on the modeled variables rather than a correction for selection on unmeasured factors.*

**Supplementary Figure S1 | Participant flow**

*Children were excluded from an analytic sample when the outcome could not be scored or when any of the 19 predictors was missing (listwise deletion). Variable-specific missingness is given in Supplementary Table S5.*

**Supplementary Methods**

Weights and design. The survey drew 10 to 20 county-level units per province with probability proportional to population size from a frame built on the sixth national population census, stratifying on the level of socio-economic development and taking geographic location within the province into account (486 units nationally); within each selected county the numbers of urban residential committees and rural village committees were fixed from that county's urban-rural population ratio and 13 committees were drawn; within each committee a fixed quota of respondents was drawn, including two children aged 3 to 6 years. The present file represents 470 of the county-level units. The weight we constructed for the survey is constant within province by urban-rural residence by child sex cells and is normalized to a mean of 1. Variance estimation treated the county-level unit as the primary sampling unit nested within the 31 provincial strata (ultimate-cluster approximation; Taylor linearization; R package survey), with t reference distributions and design degrees of freedom (sampling units minus strata). The first-stage stratum identifiers are not carried in the analysis file, so the province is used as the stratum; ignoring stratification below the province yields, if anything, conservative standard errors. Urban-rural classification is an allocation rule applied at the second stage and an attribute of the sampled committee, so it enters the models as a respondent-level covariate and not as a stratum; Supplementary Table S14 shows that stratifying on province by urban-rural instead leaves the inference unchanged. County labels supplied as free text for one province were mapped to one identifier per county before the sampling units were formed.

Decomposition. In the same analytic sample as the main model, the total association (c) is the coefficient of support in a weighted regression of the outcome on support and covariates; a-paths are the coefficients of support in regressions of each behavior on support and covariates; b-paths and the direct association (c') come from the regression of the outcome on support, the four behaviors, and covariates. Indirect components are a x b; their sum equals c minus c'. Confidence intervals are percentile intervals from 1000 bootstrap resamples that drew county-level units with replacement within each province and retained the survey weight.

Multiple imputation. Chained equations (R package mice; predictive mean matching for numeric variables, polytomous regression for parenting orientation and perceived weight), 20 imputed data sets, 10 iterations; the imputation model contained both outcomes, all 19 predictors, the survey weight, indicators for the 31 provincial strata, and the county-level means of the analysis variables, so that both the stratification and the between-cluster variation of the design were carried into the imputation. The main model was fitted in each imputed data set with the survey design and pooled by Rubin's rules (R package mitools).

Software. R 4.4.2 with packages survey, mice, mitools, sandwich, clubSandwich, relaimpo, and openxlsx. The analysis code is given in Data S1.

**Supplementary Data S1 | Analysis code**

*R code for the main analyses. Variables are coded as described in Table S8; the file path is a placeholder.*

```
library(survey); library(relaimpo); library(mice); library(mitools)
options(survey.lonely.psu = "adjust")
set.seed(20260910)

# analysis file; variables coded as in Table S8
dat <- readRDS("<analysis file>")

cont  <- c("support", "persistence", "fath_ex", "moth_ex", "fath_scr", "moth_scr",
           "coact", "equip", "commenv", "sociable", "income", "edu", "age_m")
bin   <- c("fath_ob", "moth_ob", "male", "urban")
fac   <- c("par_orient", "wt3")
preds <- c(cont, bin, fac)
covs  <- c("age_m", "male", "urban", "income", "edu")
rhs   <- paste(c(paste0("z_", cont), bin, fac), collapse = " + ")

zscore <- function(d, sds = NULL) {
  if (is.null(sds)) sds <- sapply(d[cont], sd)
  for (v in cont) d[[paste0("z_", v)]] <- (d[[v]] - mean(d[[v]])) / sds[v]
  attr(d, "sds") <- sds
  d
}
design <- function(d) svydesign(ids = ~county, strata = ~prov, weights = ~W1, data = d, nest = TRUE)

# main models
fit_main <- function(out) {
  d <- zscore(dat[complete.cases(dat[c(out, preds, "W1", "county")]), ])
  des <- design(d)
  list(d = d, des = des, fit = svyglm(as.formula(paste(out, "~", rhs)), design = des))
}
m_pa <- fit_main("PA")
m_mc <- fit_main("motor")
for (m in list(m_pa, m_mc)) {
  print(summary(m$fit, df.resid = degf(m$des)))
  print(confint(m$fit, ddf = degf(m$des)))
}

# differences between coefficients
contr <- function(m, a, b) {
  ct <- svycontrast(m$fit, setNames(c(1, -1), c(a, b)))
  est <- as.numeric(coef(ct)); se <- as.numeric(SE(ct)); df <- degf(m$des)
  c(diff = est, lo = est - qt(.975, df) * se, hi = est + qt(.975, df) * se,
    p = 2 * pt(-abs(est / se), df))
}
contr(m_pa, "z_sociable", "z_equip")
contr(m_pa, "z_coact", "z_support")
contr(m_mc, "z_support", "z_sociable")
contr(m_mc, "z_support", "z_equip")

# relative importance (LMG), covariates always in the model
lmg <- function(m, out) {
  X <- model.matrix(m$fit)[, -1]; cn <- colnames(X)
  cm <- cov.wt(cbind(m$d[[out]], X), wt = m$d$W1 / sum(m$d$W1))$cov
  colnames(cm) <- rownames(cm) <- c(out, cn)
  calc.relimp(cm, type = "lmg", rela = FALSE,
              groups = list(par_orient = grep("par_orient", cn, value = TRUE),
                            wt3 = grep("wt3", cn, value = TRUE)),
              always = c("z_age_m", "male", "urban", "z_income", "z_edu"))
}
lmg(m_pa, "PA")
lmg(m_mc, "motor")

# decomposition of the support association; county bootstrap within province
decomp <- function(d, out, meds = c("equip", "coact", "moth_ex", "fath_ex"), B = 1000) {
  est <- function(x) {
    f <- function(y, rhs) lm(as.formula(paste(y, "~", paste(rhs, collapse = "+"))), data = x, weights = W1)
    c_tot <- coef(f(out, c("support", covs)))["support"]
    a <- sapply(meds, function(md) coef(f(md, c("support", covs)))["support"])
    fb <- coef(f(out, c("support", meds, covs)))
    ind <- a * fb[meds]
    c(total = unname(c_tot), direct = unname(fb["support"]), setNames(ind, meds),
      total_ind = sum(ind), prop_ind = sum(ind) / unname(c_tot))
  }
  by_cty <- split(seq_len(nrow(d)), d$county)
  cty_prov <- split(names(by_cty), tapply(d$prov, d$county, `[`, 1))
  bs <- replicate(B, {
    idx <- unlist(lapply(cty_prov, function(cs) unlist(by_cty[sample(cs, length(cs), replace = TRUE)])))
    est(d[idx, ])
  })
  cbind(estimate = est(d), t(apply(bs, 1, quantile, c(.025, .975))))
}
decomp(m_pa$d, "PA")
decomp(m_mc$d, "motor")

# support interactions, Benjamini-Hochberg across the 12 tests
mods <- c("z_sociable", "male", "urban", "z_income", "z_edu", "z_age_m")
int <- do.call(rbind, lapply(list(PA = m_pa, motor = m_mc), function(m) {
  out <- all.vars(formula(m$fit))[1]
  t(sapply(mods, function(md) {
    f <- svyglm(as.formula(paste(out, "~", rhs, "+ z_support:", md)), design = m$des)
    ct <- summary(f, df.resid = degf(m$des))$coefficients
    ct[nrow(ct), c(1, 2, 4)]
  }))
}))
cbind(int, p_BH = p.adjust(int[, 3], "BH"))

# multiple imputation; province and county means of the analysis variables in the imputation model
ctx_v <- c("PA", "motor", "support", "equip", "coact", "fath_ex", "moth_ex", "fath_scr",
           "moth_scr", "sociable", "commenv", "income", "edu")
ctx <- as.data.frame(lapply(dat[ctx_v], function(v) {
  z <- ave(v, dat$county, FUN = function(x) mean(x, na.rm = TRUE))
  z[is.na(z)] <- mean(z, na.rm = TRUE); z
}))
names(ctx) <- paste0("ctx_", ctx_v)
imp_dat <- cbind(dat[c("PA", "motor", preds, "W1")], prov_f = factor(dat$prov), ctx)
imp_dat$persistence <- as.numeric(imp_dat$persistence)
meth <- make.method(imp_dat); meth[c("W1", "prov_f", names(ctx))] <- ""
imp <- mice(imp_dat, m = 20, maxit = 10, method = meth, seed = 20260910, printFlag = FALSE)
mi_fit <- function(out, m) {
  fits <- lapply(1:20, function(i) {
    d <- complete(imp, i); d$county <- dat$county; d$prov <- dat$prov
    svyglm(as.formula(paste(out, "~", rhs)), design = design(zscore(d, attr(m$d, "sds"))))
  })
  summary(MIcombine(fits))
}
mi_fit("PA", m_pa)
mi_fit("motor", m_mc)
```
